# Optimising *POU3F4* variant interpretation through gene-specific evidence in X-linked hearing loss

**DOI:** 10.1016/j.ebiom.2026.106318

**Published:** 2026-05-29

**Authors:** Jia Geng, Yixin Zhao, Yu Huang, Wenyu Xiong, Mingjun Zhong, Chao Wang, Xiaolu Wang, Qian Zhang, Ting Tang, Meilin Chen, Liyuan Fang, Ye Yang, Fengxiao Bu, Jing Cheng, Yu Lu, Huijun Yuan

**Affiliations:** a Department of Otolaryngology-Head and Neck Surgery, West China Hospital, Sichuan University, Chengdu, China; b Institute of Rare Diseases, West China Hospital, Sichuan University, Chengdu, China; c Department of Otolaryngology, Head and Neck Surgery, People's Hospital, Peking University, Beijing, China; d Genomics Center, Core Facility of West China Hospital, Sichuan University, Chengdu, China; e Brain Hospital of Hunan Province (The Second People's Hospital of Hunan Province), Hunan, China

**Keywords:** *POU3F4*, X-linked hearing loss, ACMG/AMP criteria, Gene-specific variant interpretation, Copy-number variation, Stop-loss variants

## Abstract

**Background:**

Standardised variant interpretation frameworks inadequately accommodate the distinct mutational and phenotypic architectures inherent to specific genes or diseases. *POU3F4* exemplifies this limitation in X-linked hearing loss, where stringent genotype–phenotype correlations and localised mutational hotspots dominate the pathogenic landscape. We sought to establish a calibrated, gene-specific evidentiary model to resolve interpretive inconsistencies for *POU3F4*.

**Methods:**

We sequenced 20,666 unrelated individuals with hearing loss and 7,258 controls using targeted panel and genome sequencing to capture single-nucleotide, indel, and structural variants in *POU3F4*. By integrating clinical phenotyping, paralog-based residue constraint modelling, and Bayesian likelihood-ratio analysis, we redefined evidence criteria for this gene. Representative stop-loss alleles underwent functional characterisation to elucidate their molecular consequences.

**Findings:**

Genomic analysis identified 123 distinct *POU3F4* variants, 87 of which were classified as pathogenic (P) or likely pathogenic (LP). Structural variants accounted for 29 of these P/LP variants, with breakpoints enriched in repeat-dense sequence across the locus. Incomplete partition type III cochlear malformation showed near-diagnostic coupling to *POU3F4*, with 96.4% of affected cases carrying P/LP variants, supporting escalation of PP4 to strong evidence. P/LP missense variants were significantly concentrated at paralog-conserved residues, and Bayesian modelling of this constraint produced a positive likelihood ratio of 33.0 (95% confidence interval 10.68–102.28), consistent with moderate-strength hotspot evidence of PM1. Applying the calibrated rules reclassified 17 single nucleotide variants or small indels and resolved 16 of 34 variants of uncertain significance. Functional assays demonstrated that stop-loss variants generate hydrophobic C-terminal extensions that destabilise POU3F4, driving nuclear depletion and loss of transcriptional activity. Enforced nuclear targeting restored localisation but not transcription, implicating protein instability rather than impaired nuclear import as the dominant mechanism.

**Interpretation:**

*POU3F4* pathogenicity is defined by a highly specific cochlear signature and profound topological constraint. Quantitative calibration of these features substantially refines diagnostic resolution in *POU3F4-*mediated hearing loss. The convergence of genomic modelling and functional validation establishes a calibrated evidentiary framework for consistent variant classification.

**Funding:**

This work was supported by National Natural Science Foundation of China (82530036, 82471889, 82171836), National Key Research and Development Program of China (2024YFC3405704), West China Hospital, Sichuan University 1.3.5 Project for Disciplines of Excellence grant ZYJC20002, Sichuan Provincial Natural Science Foundation (2024NSFSC0648).

**Research in context**

**Evidence before this study**

*POU3F4* is an X-linked transcription factor responsible for DFNX2-associated hearing loss, classically characterised by incomplete partition type III (IP-III) malformation and risk of perilymphatic gusher. Although numerous pathogenic variants have been reported, variant interpretation remains challenging due to reliance on generalised ACMG/AMP guidelines without gene-specific calibration. Previous studies have described recurrent deletions, missense variants within POU functional domains, and occasional neurodevelopmental features; however, the quantitative strength of genotype–phenotype correlations, the contribution of paralog conservation, and the mechanistic basis of stop-loss variants have not been systematically evaluated in large cohorts. Furthermore, the real-world impact of gene-specific refinement on reducing VUS has not been formally quantified.

**Added value of this study**

Leveraging a nationwide cohort of 20,666 unrelated hearing loss cases, we performed a comprehensive systematic analysis of *POU3F4*. We quantitatively calibrated phenotype specificity (PP4) based on the high concordance between IP-III and pathogenic variants, and established paralog-conserved residues within functional domains as a PM1 hotspot supported by Bayesian enrichment analysis. Application of this gene-specific framework resolved 47.1% of VUS among SNVs/indels. We further demonstrate that stop-loss variants generate highly hydrophobic C-terminal extensions that reduce protein stability, impair nuclear localisation, and abolish transcriptional activity. Forced nuclear targeting partially restored localisation but failed to rescue transcriptional function, protein instability contributes substantially to the functional impairment of stop-loss variants. In addition, we show that deletion breakpoints are frequently located within repeat-rich genomic regions, refining interpretation of upstream noncoding CNVs.

**Implications of all the available evidence**

Taken together with previous work, our findings improve the reliability of genetic testing for families with suspected *POU3F4*-related hearing loss and reduce the number of variants labelled as uncertain. More accurate classification supports clearer diagnosis, prognosis, and reproductive counselling for affected families. By revealing how different types of genetic changes disrupt the POU3F4 protein and inner ear development, this study also deepens understanding of the biological mechanisms underlying X-linked deafness. Beyond this single gene, the framework we propose illustrates how large patient cohorts, population data, and functional experiments can be combined to optimise gene-specific criteria for other hereditary diseases, thereby strengthening the clinical impact of genomic medicine and making it more relevant to everyday patient care.

## Introduction

The integration of high-throughput sequencing into clinical genetics has transformed variant interpretation, yet the reliance on generalised frameworks such as the 2015 ACMG/AMP guidelines exposes limitations in genetically heterogeneous conditions.^1^ Inconsistent classifications often arise when gene-specific knowledge is lacking. To address these gaps, ClinGen has developed disease- and gene-specific rules, including frameworks for *MYH7* in inherited cardiomyopathies and germline *APC* variants in hereditary colorectal cancer.^2^^,^^3^ These advances underscore the need for tailored approaches to reduce uncertainty in variant pathogenicity, particularly in hearing loss (HL), where extreme genetic heterogeneity complicates clinical interpretation.

Hearing loss, the most prevalent congenital sensory impairment, affects 1.3–2.7 per 1000 live births and involves more than 200 causative genes. The expanding use of next-generation sequencing has increased the identification of variants of uncertain significance (VUS), highlighting the necessity of robust gene–phenotype correlation frameworks. Within this landscape, *POU3F4* (Xq21.1) is a prototypical gene for X-linked mixed or sensorineural hearing loss. Male hemizygotes typically present with pathognomonic incomplete partition type III (IP-III) malformations, with enlarged vestibular aqueducts (EVA) observed in approximately 50% of cases,^4^ as well as perilymphatic gusher during cochlear implantation.^5^ Female carriers, on the other hand, may experience progressive hearing decline despite preserved cochlear anatomy.^6^ The gene encodes a POU domain–containing transcription factor, with bipartite DNA-binding domains (POU-specific and POU-homeodomain) that are highly conserved and functionally critical.^7^

To date, more than 120 *POU3F4* variants have been implicated in nonsyndromic hearing loss, encompassing single-nucleotide variants (SNVs), small insertions/deletions (indels), and large genomic deletions that disrupt both coding sequences and upstream regulatory elements (Supplemental Tables S1 and S2). Notably, multiple pathogenic deletions spanning 500 kb to 1 Mb upstream of *POU3F4* have been reported. This gene desert contains four inner-ear–related enhancers of cis-regulatory elements (CREs, HCNR81675, HCNR81728, HCNR82478, and HCNR82637) validated in vivo in Xenopus and zebrafish.^8^ Despite these advances, variant interpretation remains challenging. Approximately 16.6% of *POU3F4* variants catalogued in ClinVar continue to be classified as VUS, with missense substitutions accounting for the majority of unresolved cases. Such variants often lack robust functional evidence or consistent genotype–phenotype correlations, leading to uncertainty in pathogenicity assessment. Addressing these gaps requires systematic efforts to establish gene-specific evidence criteria, integrate large-scale cohort data, and incorporate functional validation to refine interpretation frameworks.

Here, leveraging the China Deafness Genetics Consortium (CDGC) cohort of 20,666 unrelated individuals with hearing loss, we conducted the largest systematic analysis of *POU3F4* to date. By integrating molecular spectrum data, quantitative genotype–phenotype correlations, and structural and functional analyses, we refined the gene-specific application of ACMG/AMP criteria for *POU3F4*. Specifically, we calibrated IP-III as strong PP4 evidence based on high locus specificity, identified paralog-conserved residues within functional domains as a PM1 hotspot supported by Bayesian enrichment analysis, and characterised the repeat-rich genomic architecture underlying large deletions, and explored the functional effects of stop-loss variants. Together, these findings establish a data-driven framework for *POU3F4* variant interpretation and enhance diagnostic precision in X-linked hearing loss.

## Methods

### Study cohort

The CDGC is a nationwide collaborative project established in 2013 to investigate the genetic basis of hearing loss across mainland China. Between 2013 and 2022, individuals with hearing impairment were prospectively recruited from special education schools, rehabilitation centres, and tertiary hospitals across multiple regions of China. Both male and female participants were included in this study. Sex was recorded based on self-report or clinical records at the time of recruitment. Participants underwent standardised clinical evaluation, including medical history review, physical examination, and audiometric testing. Individuals with conductive hearing loss, presbycusis, unilateral hearing loss, or mild hearing impairment (pure tone average ≤40 dB) were excluded. A subset of participants underwent a temporal bone radiologically test to diagnose inner ear deformity. After quality control and removal of related individuals, 20,666 unrelated individuals with disabling hearing loss were included in the study. Controls consisted of 7,258 unrelated adults without self-reported hearing loss. The cohort predominantly comprised early-onset, severe-to-profound, and nonsyndromic cases. Further details on recruitment criteria and demographic characteristics are provided in the Supplemental Methods.

### Genetic sequencing and variant detection

Genomic DNA was extracted from the peripheral blood samples of all subjects and initial variant screening was performed using a multiplex SNPscan assay targeting common deafness-associated variants. Participants without a molecular diagnosis underwent targeted sequencing of 157 hearing loss–related genes using the CDGC-HL capture panel. Libraries were prepared according to standard protocols and sequenced on an Illumina platform with paired-end reads. Cases remaining genetically undiagnosed after panel testing were further analysed by short-read genome sequencing using the DNBSEQ-T7 platform (BGI, Shenzhen, China). Sequencing reads were aligned to the human reference genome (GRCh37/hg19) using Burrows–Wheeler Aligner, and variant calling was performed following Genome Analysis Toolkit best practices. Structural variants were detected using established algorithms. For one unresolved case with radiologically confirmed incomplete partition type III (IP-III), long-read sequencing (Oxford Nanopore Technologies) was performed to further evaluate structural variants. Detailed software versions and parameters are provided in the Supplemental Methods.

### Variant inclusion criteria

**Fig. 1:**
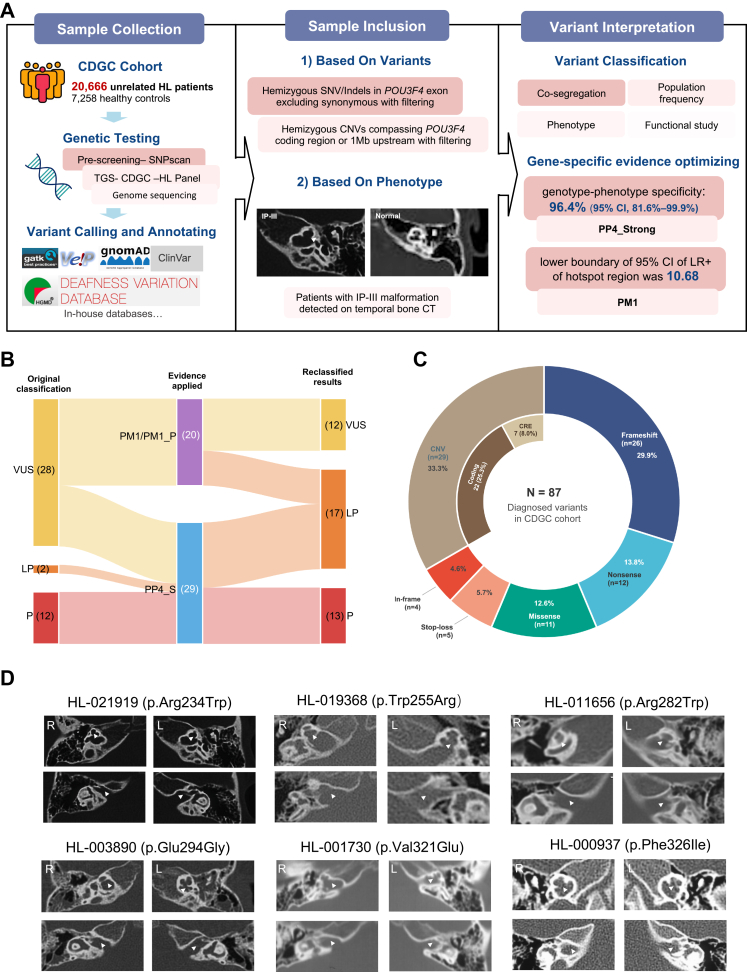
**Genetic and phenotypic analysis pipeline for *POU3F4*-related hearing loss.** (A) Study workflow for 20,666 unrelated hearing loss patients and 7258 controls from the CDGC cohort. Genetic testing included SNPscan pre-screening, targeted sequencing (TGS), and genome sequencing. Variants were classified using ACMG/AMP criteria and gene-specific evidence, incorporating co-segregation, population frequency, phenotype correlation, and functional assays. (B) Reclassification of *POU3F4* variants with gene-specific criteria. Reclassification of original variants (VUS, LP, P) based on quantitative genotype–phenotype correlation and Bayesian calibration. The flowchart depicts the reclassification of the 42 variants that met the newly defined gene-specific criteria (PP4_Strong and/or PM1/PM1_Supporting), which represent a subset of the total 79 SNVs/indels identified in the cohort. (C) Breakdown of 87 diagnosed *POU3F4* variants in the CDGC cohort. Predominantly CNVs (33.3%) and frameshift mutations (29.9%). (D) Temporal bone CT images of patients with novel pathogenic *POU3F4* variants, showing IP-III malformation with absent modiolus and enlarged internal auditory canal. CDGC, Chinese Deafness Genetics Consortium; HL, hearing loss; TGS, targeted gene sequencing; SNV, single nucleotide variant; indels, insertions/deletions; CNV, copy number variant; IP-III, incomplete partition type III; CT, computed tomography.

Hemizygous SNVs/indels in *POU3F4* (excluding synonymous variants) and copy-number variants affecting the coding region or upstream regulatory interval were included based on allele frequency thresholds and quality metrics (Fig. 1A). Variants were filtered based on sequencing quality metrics and allele frequency thresholds, excluding variants with a minor allele frequency >0.005 in population databases (gnomAD v4.1.0) or in-house control datasets. Population stratification between cases and controls was assessed using principal component analysis (PCA), which demonstrated no substantial population structure bias. To minimise potential bias, only unrelated individuals were included in the analysis. Individuals with missing key variables were excluded from specific analyses. The study size was determined by the availability of participants in the CDGC cohort. Given the X-linked nature of POU3F4-related hearing loss, sex was considered in the interpretation of genetic variants and clinical phenotypes. Detailed filtering criteria and annotation procedures are provided in the Supplemental Methods.

### Construction of high-confidence reference sets

To enable quantitative calibration of gene-specific criteria, high-confidence pathogenic reference set and non-pathogenic reference sets were constructed prior to application to the study cohort to avoid circularity. The pathogenic reference set included variants with strong prior evidence of pathogenicity. Predicted loss-of-function variants truncating the protein before the end of the POU homeodomain (amino acid 340) were included, consistent with the established loss-of-function disease mechanism. Non-truncating variants were included only if identified in individuals with radiologically confirmed IP-III. Pathogenic or likely pathogenic CNVs were included according to ClinGen CNV interpretation guidelines.

A non-pathogenic reference set was constructed from hemizygous missense variants observed in gnomAD (v4.1.0), representing variants unlikely to be disease-causing under an X-linked model.

### Quantitative calibration of PP4 and PM1

IP-III was defined by characteristic temporal bone CT findings. Genotype–phenotype concordance was quantified using exact binomial confidence intervals (Clopper–Pearson method).

Missense enrichment within functional domains and paralog-conserved residues was assessed by comparing pathogenic and non-pathogenic reference sets. Positive likelihood ratios (LR+) were calculated using established Bayesian thresholds, employing the following formulae^9^:

$${LR}^{+}=\frac{\left(\#P/LP\;variants\;in\;hotspot\;region\right)/\left(\#P/LP\;variants\;in\;the\;gene\right)}{1\;-\;\left(\#NonP\;variants\;outside\;hotspot\;region\right)/\left(\#NonP\;variants\;in\;the\;gene\right)}$$

where # is the number of variants.

Calibration of PM1 and PP4 was performed prior to application to the study cohort to avoid circularity.

### Development of the POU3F4 gene-specific interpretation framework

Based on 2015 ACMG/AMP guidelines^1^ and specifications from the ClinGen Hearing Loss Variant Curation Expert Panel (HL VCEP).^9^ gene-specific refinements for *POU3F4* were developed through two complementary approaches: 1) principle-based adaptation of the ACMG/AMP framework informed by previously published gene-specific refinements, with adjustments tailored to the inheritance pattern, disease mechanism, and molecular characteristics of *POU3F4*; 2) data-driven calibration of PM1 and PP4 based on quantitative analyses derived from the CDGC cohort.

Final variant classification followed the ACMG/AMP combinatorial framework, with categories assigned strictly according to predefined evidence–strength combinations. The finalised *POU3F4*-specific criteria were subsequently applied to all variants identified in the study cohort.

### Functional assays and structural modelling

Wild-type (WT), stop-loss and rescued *POU3F4* expression constructs were generated and transfected into HEK293T and HeLa cells. Protein expression levels were assessed by Western blot, and subcellular localisation was evaluated by immunofluorescence microscopy. Nuclear and cytoplasmic distribution of POU3F4 proteins was quantified based on fluorescence intensity measurements. To investigate whether impaired nuclear localisation contributes to functional defects, rescue constructs incorporating an exogenous nuclear localisation signal (NLS) were generated. Transcriptional activity was assessed using dual-luciferase reporter assays, with firefly luciferase activity normalised to Renilla luciferase as a control for transfection efficiency.

To explore the structural impact of stop-loss variants, three-dimensional models of WT and extended POU3F4 proteins were generated using AlphaFold2. Models were superimposed based on the POU domain backbone, and spatial relationships between C-terminal extensions and the predicted nuclear localisation signal (NLS, residues 275–286) were examined. Detailed experimental procedures, antibody information, and modelling parameters are provided in the Supplemental Methods.

### Ethics

This study was approved by the Ethics Committee of West China Hospital, Sichuan University (approval no. 2021 Audit (190)) and the Ethics Committee of Southwest Hospital (approval no. Scientific Research 2015 (56)). Written informed consent was obtained from all participants or their guardians, in accordance with the principles of the Declaration of Helsinki.

### Statistical analysis

Statistical analyses were performed using R software (version 4.4.2). Data are presented as mean ± standard deviation (SD) or as proportions, as appropriate. Exact binomial confidence intervals (Clopper–Pearson method) were used to estimate genotype–phenotype concordance. Positive likelihood ratios (LR+) and corresponding 95% confidence intervals were calculated based on a Bayesian framework. Odds ratios (ORs) and 95% confidence intervals were calculated for case–control analyses, with continuity correction applied where appropriate. Fisher's exact test was used for case–control enrichment analyses where appropriate. For functional assays, comparisons between two groups were performed using two-tailed Student's t-test, and comparisons involving multiple conditions were analysed using two-way analysis of variance (ANOVA) followed by Šidák's multiple-comparison test where appropriate. A P-value <0.05 was considered statistically significant. The study sample size was determined by the availability of participants in the CDGC cohort. No formal randomisation was applied in this observational study. For functional experiments, data quantification was performed in a blinded manner where applicable.

### Role of funders

The funders had no role in study design, data collection, data analysis, data interpretation, or writing of the report.

## Results

### Cohort overview and quantitative calibration of phenotype specificity

A total of 20,666 unrelated individuals with hearing loss and 7258 unaffected controls were enrolled to evaluate the contribution of *POU3F4* to hearing loss. After initial filtering, 123 unique variants were identified in 129 affected individuals, including 79 SNVs/indels and 44 CNVs. Among these, 56 SNVs/indels (Supplemental Table S3) and 29 CNVs (Supplemental Table S4) met criteria for inclusion in the high-confidence pathogenic reference set. An additional 88 SNVs/indels (Supplemental Table S1) and 33 CNVs (Supplemental Table S2) extracted from published literature satisfied the same inclusion criteria.

28 patients with radiologically confirmed incomplete partition type III (IP-III), of which 27 harboured high-confidence pathogenic variants (Fig. 1D, Supplemental Figure S1), yielding a genotype–phenotype concordance of 96.4% (95% CI: 81.6–99.9). Despite the modest sample size, the lower bound of the confidence interval remained high, supporting strong locus specificity. One female patient with IP-III remained genetically unresolved after comprehensive short- and long-read genomic analyses (Supplemental Figure S2), suggesting that alternative genetic etiologies for IP-III are uncommon. Among the 88 individuals carrying high-confidence pathogenic variants in the CDGC cohort, 27 had radiologically confirmed IP-III. No additional major inner ear malformation pattern distinct from IP-III was documented in this group. Among them, 22 individuals with temporal bone imaging of sufficient quality to evaluate the vestibular aqueduct, EVA was present in 10 cases with 7 bilateral and 3 unilateral. The EVA phenotype was consistent with prior reports,^4^ characterised by enlargement predominantly at the mid-portion and cochlear end of the vestibular aqueduct (Supplemental Figure S3).

### Domain enrichment and Bayesian calibration of missense variants

**Fig. 2:**
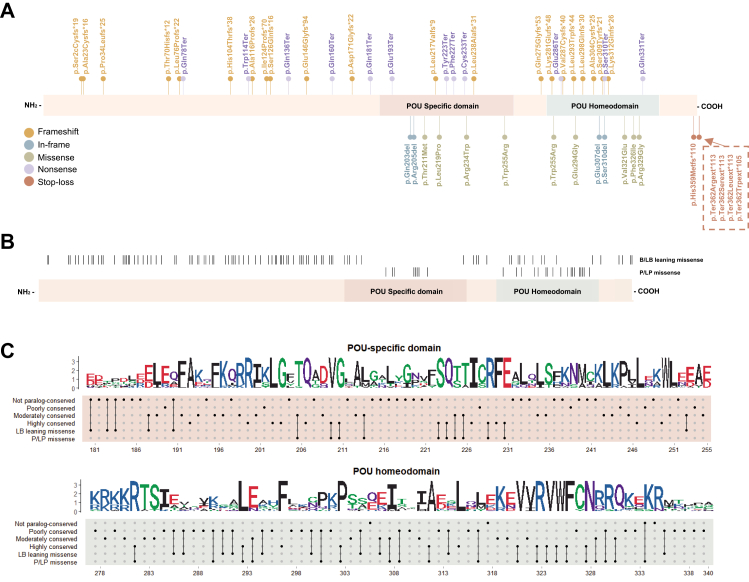
**Distribution of pathogenic variants in *POU3F4*.** (A) Distribution of pathogenic SNVs in the POU3F4 protein structure. Variants are colour-coded by type: frameshift (red), in-frame (purple), missense (orange), nonsense (yellow), and stop-loss (brown). (B) Schematic of the POU3F4 protein showing the locations of LB-leaning missense (hemizygous missense variants from gnomAD v4.1.0), and P/LP missense variants identified in this study and curated from the literature. Tick marks denote variant positions along the protein. (C) Sequence-logo representation of paralogous multiple-sequence alignments for the POU-specific domain (top) and POU-homeodomain (bottom) across POU-family proteins. Letter height reflects information content (bits), indicating residue conservation. Amino acids are coloured by physicochemical class (acidic, red; basic, blue; hydrophobic, black; neutral, purple; polar, green). The dot matrix beneath each logo summarises paralog-conservation at each residue (not, poorly, moderately, or highly conserved) and maps the positions of LB-leaning missense variants and P/LP missense variants to the corresponding aligned residues.

Using the high-confidence pathogenic missense reference set (n = 32; Supplemental Table S5), we evaluated the spatial distribution of missense variants across *POU3F4*. All 32 pathogenic missense variants were located within either the POU-specific domain (amino acids 181–255) or the POU homeodomain (amino acids 278–340). In contrast, 122 hemizygous missense variants identified in male individuals from gnomAD (v4.1.0) were used as a non-pathogenic reference set, of which 85.2% (104/122) were located outside these two functional domains (Fig. 2A and B) We previously demonstrated missense variants located in *POU3F4* domains fulfilled the supporting level.^10^

Because *POU3F4* amino acid sequences are highly conserved across species (Supplemental Figure S4), orthologue-based conservation analysis provides limited resolution for distinguishing functionally critical residues. We therefore performed paralog conservation analysis across 15 POU family proteins to refine residue-level constraint and assess enrichment of pathogenic variants at conserved positions. Specifically, 81.3% (26/32) of pathogenic missense variants affected highly or moderately conserved residues, compared with only 2.5% (3/122) of variants in the non-pathogenic reference set. The LR+ for pathogenicity at conserved residues was 33 (95% CI: 10.68–102.28). Importantly, the lower bound of the confidence interval exceeded the moderate Bayesian threshold (LR+ ≥4.3) defined by Tavtigian et al.,^9^ supporting moderate-level evidence for PM1 calibration (Fig. 2C, Supplemental Figure S5, Table 1).

**Table 1:** Distribution of pathogenic and non-pathogenic varians in relation to position of POU3F4 hotsopts.

Presence in	Pathogenic	Not pathogenic	Positive likelihood ratio (LR+)	95% CI
Highly and moderatedly paralog-conserved amino acids	26	3	33	10.68–102.28
Lowly and poorly paralog-conserved amino acids	6	119	–	–

Carrier-based case–control enrichment analyses were performed using 20,666 unrelated hearing loss cases and 7,258 controls. Predicted loss-of-function variants (frameshift and nonsense) were observed in 39 cases and in none of the controls (P = 1.10 × 10^−5^, Fisher's exact test). Using a continuity correction, the estimated odds ratio (OR) was 27.8 (95% CI: 1.71–452.3), strongly supporting a loss-of-function disease mechanism for *POU3F4*. In contrast, missense variants as a class were not significantly enriched in cases compared with controls (OR = 1.61, 95% CI: 0.71–3.64; P = 0.36, Fisher's exact test). Similarly, missense variants located within the POU functional domains did not show significant enrichment (OR = 1.58, 95% CI: 0.34–7.32; P = 0.74, Fisher's exact test). However, variants affecting paralog-conserved residues showed a directional enrichment (OR = 3.16, 95% CI: 0.39–25.3; P = 0.27, Fisher's exact test). Although statistical significance was not reached, likely due to the low carrier frequency of missense variants, these observations suggest that pathogenic missense substitutions may preferentially affect evolutionarily constrained residues rather than being broadly enriched across all missense variation (Supplemental Table S6).

PCA of genome-wide variants demonstrated no evident clustering between cases and controls, indicating minimal population stratification. Ethnicity-stratified PCA revealed expected population structure but showed good mixing of cases and controls within clusters, supporting the validity of the enrichment analysis (Supplementary Figure S6).

### Impact of gene-specific calibration on SNV/indels classification

Among the 28 ACMG/AMP evidence criteria, 13 were modified with respect to their utilisation and/or strength level for *POU3F4* (Supplemental Table S7, (Supplemental Appendix). Two criteria (PM3 and BS2) were deemed not applicable due to the X-linked inheritance pattern. Four additional criteria (PP2, PP5, BP1, and BP6) had previously been classified as not applicable by the ClinGen HL VCEP. Notably, PM1 and PP4 were re-specified based on quantitative analyses derived from the CDGC cohort, providing gene-specific calibration of domain enrichment and genotype–phenotype concordance.

Application of the *POU3F4* gene-specific criteria led to quantifiable reclassification within the CDGC cohort. Prior to applying the recalibrated guidelines, among the 79 identified SNVs/indels, 20 were classified as P, 24 as LP, 34 as VUS, and 3 as LB. After applying the new criteria based on CDGC cohort data, 53.2% (42/79) variants met at least one of the recalibrated criteria (PP4_Strong and/or PM1/PM1_Supporting). Specifically, 8.9% (7/79) of missense variants simultaneously fulfilled both PP4_Strong and PM1/PM1_Supporting, all of which were upgraded from VUS to LP. An additional 27.8% (22/79) of variants met PP4_Strong alone, of which 9 underwent conclusion changes: 8 were upgraded from VUS to LP (including three missense, two stop-loss, and three in-frame variant), and 1 in-frame variant was upgraded from likely pathogenic to pathogenic due to sufficient weighted evidence. The remaining 13 variants did not change classification. Of these, 12 were already classified as pathogenic (8 frameshift and 4 nonsense variants), for which the additional phenotype evidence did not alter their final category. One stop-loss variant remained classified as likely pathogenic. In contrast, 16.5% (13/79) of missense variants fulfilled PM1 or PM1_Supporting alone. Among these, only 1 variant was reclassified from VUS to LP, while the remaining 12 variants did not meet the threshold for reclassification despite domain-based enrichment support. These data demonstrate the impact of the gene-specific framework in reclassifying variants, with a significant proportion of VUS resolved to clinically relevant categories (Fig. 1B, Supplemental Table S3).

Overall, incorporation of gene-specific calibration resulted in reclassification of 17 out of 79 SNVs/indels (21.5%). Importantly, 16 of these represented clinically meaningful upgrades from VUS to LP, corresponding to resolution of 47.1% (16/34) of VUS identified among SNVs/indels. The majority of reclassifications (15/16, 93.8%) were driven by strengthened phenotype specificity (PP4_Strong), highlighting IP-III–based calibration contributes substantially to diagnostic improvement.

### Characterisation and implications of *POU3F4*-related CNVs

**Fig. 3:**
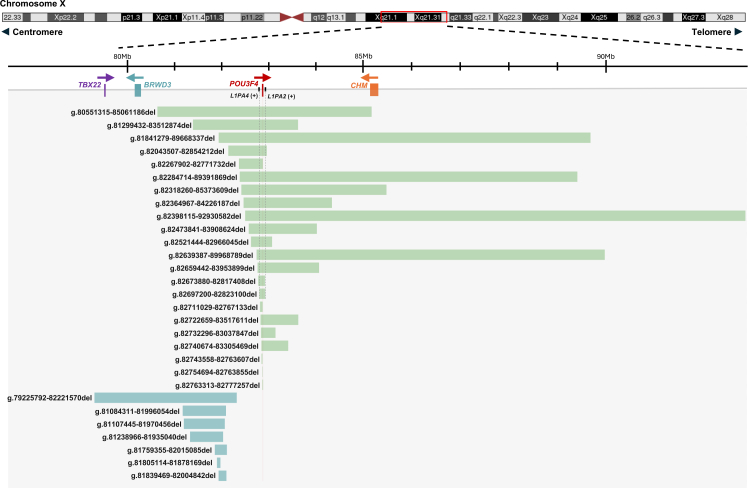
**Genomic structure and mapping of *POU3F4*-related deletions in the CDGC cohort.** Schematic of the X chromosome region harbouring *POU3F4*. Deletion events are represented as horizontal bars: coding deletions affecting *POU3F4* are shown in green, non-coding deletions in blue. Genomic coordinates are listed on the left. Genes with ClinGen haploinsufficiency scores of 3 are highlighted: *TBX22* (purple), *BRWD3* (blue), and *CHM* (orange). Arrows indicate transcriptional direction. (+) denotes strand orientation of *L1PA4* and *L1PA2* repeat elements.

We identified 29 P/LP large deletions (>50 bp) in 30 patients, including 22 spanning coding regions of *POU3F4* and 7 located upstream affecting CREs (Fig. 3). Breakpoints were determined by Sanger sequencing or BAM file review in IGV, except in one patient (HL-023624) (Supplemental Figure S7). Upstream deletions varied in size, ranging from 500 kb to 1 Mb upstream of *POU3F4*, and overlapped regions containing previously reported enhancers. Several deletions coincided with the minimal overlapping deletion interval described in prior studies (Supplemental Figure S8). To refine interpretation of upstream noncoding deletions, we annotated each CNV for overlap with four experimentally validated enhancers and scored them according to the ACMG/ClinGen 2020 CNV interpretation standards (Supplemental Table S4).

RepeatMasker analysis of flanking sequences showed that the deletion in HL-023624 (X:g.82697250–82823100del) was mediated by LINE-1 repeats (*L1PA4* and *L1PA2*) (Supplemental Figure S9). Thirteen additional deletions had breakpoints within repetitive elements without significant homology, and 11 had one breakpoint mapping to repetitive sequences (Supplemental Table S10). These findings indicate that deletion breakpoints are frequently located within repeat-rich regions of *POU3F4*, though no single dominant recombination mechanism could be established.

In addition to *POU3F4*, three OMIM genes with haploinsufficiency scores of 3 were encompassed in some deletions: *TBX22* (X-linked cleft palate and ankyloglossia), *BRWD3* (X-linked intellectual disability), and *CHM* (choroideremia). Five patients had deletions spanning the entire *CHM* gene. At the time of clinical evaluation (ages 4–18 years), none of these patients reported night blindness or visual field deficits, and no ophthalmologic findings were available. Given the progressive nature of choroideremia, early symptoms such as nyctalopia, which usually emerge in preteens, may not have been recognised in this age range. One patient (HL-012590) carried a deletion spanning *TBX22* and *BRWD3*. At age 7, no cleft palate, craniofacial anomalies, or signs of intellectual disability were found, and the guardian declined further diagnostic assessments, limiting evaluation of extra-auditory phenotypes.

### Characterisation of *POU3F4* dignosed patients in CDGC cohort after re-classification

Among the 123 variants in CDGC cohort, including both SNVs/Indels and CNVs, 87 (70.7%) variants detected in 91 unrelated male were classified as P/LP, 33 (26.8%) as VUS, and 3 (2.4%) as likely benign. Truncating variants predominated among P/LP SNVs/indels, while CNVs accounted for 33.3% (29/87) of P/LP variants, including 22 coding and 7 CREs alterations (Fig. 1C). Among the 91 diagnosed probands, the mean age at audiologic evaluation was 9.2 ± 7.4 years. 67 individuals were classified as having SNHL, 3 as mixed hearing loss, and detailed audiologic subtype information was unavailable for 21 individuals (Supplemental Table S8). The majority exhibited severe-to-profound impairment. In addition, eight heterozygous females from six pedigrees were also identified, demonstrating variable expressivity consistent with X-linked inheritance (Supplemental Table S9). Five individuals were reported to have neurodevelopmental features, including developmental delay, intellectual disability, or autism spectrum disorder.

### Functional effects of stop-loss variants

**Fig. 4:**
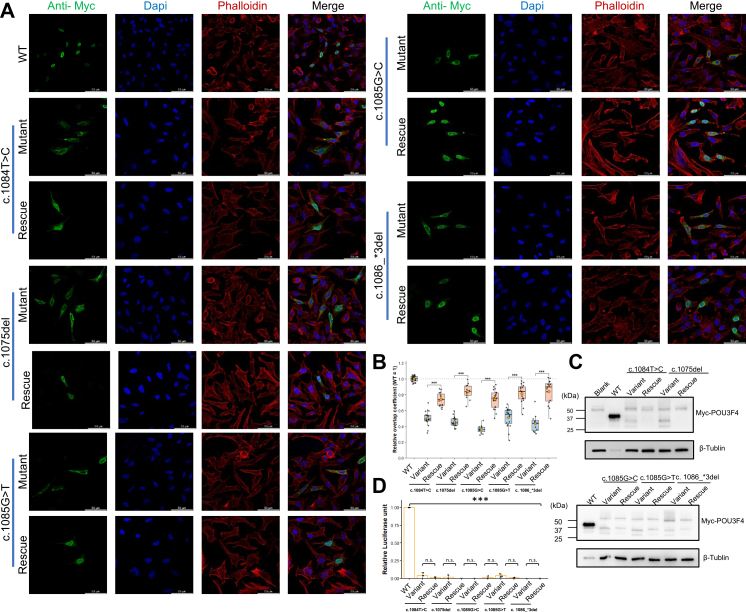
**Functional impact of *POU3F4* stop-loss variants and rescue effect.** (A) Immunofluorescence staining showing the subcellular localisation of wild-type (WT) and stop-loss variant POU3F4 proteins, with and without NLS-rescue. The localisation of proteins was assessed using anti-Myc (green), DAPI (blue), and Phalloidin (red) staining. The rescue constructs partially restored nuclear localisation of the variant proteins. (B) Quantification of nuclear localisation from immunofluorescence data. Each dot represents a single cell. Data were derived from three independent biological replicates, with at least 12 cells analysed per condition across experiments. Statistical comparisons between each variant and its matched rescue construct were performed using two-way ANOVA followed by Šidák's multiple-comparison test. Data are presented as mean ± SD. The WT nuclear/cytoplasmic ratio was set as 1.0 for normalisation. (C) Western blot analysis showing the expression of Myc-tagged *POU3F4* variants in HEK293T cells. Proteins were detected using an anti-Myc antibody. β-tubulin was used as a loading control. (D) Quantification of dual-luciferase reporter assay results, showing transcriptional activity of the *POU3F4* variants and their corresponding rescue constructs. ∗∗∗P < 0.001 (two-way ANOVA with Šidák's multiple-comparison test), n.s. = not significant.

To assess whether stop-loss variants affect *POU3F4* protein expression, HEK293T cells were transfected with either WT or five stop-loss variants (c.1084 T > C, c.1075 del, c.1085G > T, c.1085G > C, and c.1086_∗3 del), followed by Western blot analysis at 12, 24, and 48 h post-transfection. Under equal transfection conditions (500 ng plasmid), protein expression levels were consistently higher for WT compared to all five variants at each time point. Protein abundance peaked at 24 h for both WT and variant constructs, then decreased by 48 h. When transfected at both 250 ng and 500 ng plasmid amounts, WT protein levels remained consistently higher than those of the variants (Supplemental Figure S10). We next investigated the subcellular localisation of WT and stop-loss variant proteins. While WT proteins were normally localized to the nucleus, stop-loss variants failed to localise there in HeLa cells. Instead, variant proteins accumulated in the cytoplasm (Fig. 4A).

Although five stop-loss variants were identified, they produced only two distinct C-terminal extension peptides. Both extensions were highly hydrophobic, with Kyte–Doolittle mean hydropathy scores of 0.93 (Extension 1; 112 aa) and 1.10 (Extension 2; 105 aa), and hydrophobic residues constituting 54.5% and 59.0% of the sequences, respectively. Structural models further suggested that the extensions approach the predicted NLS (residues 275–286), with minimum Cα–Cα distances of 14.35 Å (Mut 1) and 17.73 Å (Mut 2), suggesting a plausible indirect interference with nuclear import. These data indicate that the hydrophobic extensions may perturb protein conformation and/or promote cytoplasmic retention, reducing nuclear localisation despite an intact NLS sequence (Supplemental Figure S11).

To further assess whether defective nuclear import was the primary driver of functional impairment, we introduced an exogenous SV40 NLS upstream of the Myc tag. Addition of the SV40 NLS did not alter protein amount, as NLS-rescue constructs exhibited expression levels comparable to those of the corresponding stop-loss variants at 24 h post-transfection (Fig. 4C). Immunofluorescence analysis demonstrated partial restoration of nuclear localisation, with approximately half of the residual variant protein redistributed to the nucleus, while the remainder persisted in the cytoplasm (Fig. 4A). Quantification of the nuclear/cytoplasmic distribution of POU3F4 proteins revealed that stop-loss variants exhibited significantly reduced nuclear localisation compared to WT controls. The relative overlap coefficient of variant proteins with the nuclear region was lower, suggesting a stronger cytoplasmic accumulation. Rescue constructs showed partial restoration of nuclear localisation, though the proteins were still predominantly cytoplasmic. The data indicate that the nuclear localisation defect observed in stop-loss variants can be partially rescued by the N-terminal NLS, but full functional rescue remains unachieved. However, dual-luciferase assays showed that SV40 NLS-tagged variants exhibited no significant recovery of transcriptional activity compared with the original stop-loss constructs (Fig. 4D), indicating although impaired nuclear localisation contributes to the phenotype, restoration of nuclear targeting alone is insufficient to rescue function. These findings suggest that protein instability contributes substantially to the functional impairment of stop-loss variants.

## Discussion

In this study, we established and validated a large-scale, gene-specific interpretation framework for *POU3F4* based on the CDGC hearing loss cohort, integrating comprehensive variant profiling, quantitative phenotype calibration, CNV characterisation, and functional analysis of stop-loss variants.

By analysing 20,666 individuals with hearing loss and 7,258 controls, we curated high-confidence pathogenic reference sets for both SNVs/indels and CNVs. We demonstrated a strong genotype–phenotype concordance between *POU3F4* variants and IP-III malformation, providing quantitative support for phenotype-driven evidence strengthening (PP4_Strong). Through domain enrichment and paralog conservation analyses, we also calibrated the PM1 criterion at a moderate level, establishing objective thresholds for interpreting missense variants. Incorporating these gene-specific specifications led to clinically meaningful reclassification of 21.5% of SNVs/indels, resolving 47.1% of previously classified VUS. Although predicted loss-of-function variants showed strong case enrichment, missense variants did not reach statistical significance in the case–control analysis. This pattern likely reflects both limited statistical power and biological heterogeneity among missense variants. The carrier frequency of *POU3F4* missense variants is very low in both cases and controls, reducing the ability of burden analyses to detect modest enrichment. In addition, many missense substitutions are likely functionally tolerated, resulting in the coexistence of benign and pathogenic variants that dilute the overall signal. Importantly, pathogenic missense variants were strongly concentrated at paralog-conserved residues within the POU functional domains, producing a high positive likelihood ratio that exceeds the Bayesian threshold for moderate evidence. Thus, missense pathogenicity in *POU3F4* appears to be driven primarily by disruption of specific functionally constrained residues rather than by a general increase in missense burden, supporting the application of PM1 despite the absence of population-level enrichment.

These gene-specific adaptations align with the established ACMG/AMP–ClinGen framework and represent a recalibration rather than the introduction of entirely new interpretation standards. Modifications to evidence strength were made based on empirical data, such as the high genotype–phenotype concordance for IP-III (supporting PP4_Strong) and statistically significant domain enrichment with a likelihood ratio exceeding the Bayesian thresholds for PM1 (moderate level). This method is consistent with other ClinGen gene-specific implementations, such as for *MYH7*,^2^ *APC*,^11^ *FBN1*,^12^ and *SLC6A8*,^13^ where evidence strength was adjusted based on measurable effect sizes and consistent mechanisms. These refinements, particularly those driven by inheritance patterns, are aligned with precedents in X-linked disorders and ensure the methodology's consistency and potential applicability to other monogenic hearing loss genes. However, the quantitative thresholds derived here are tailored to *POU3F4* and should not be extrapolated to other genes without independent validation. Despite this, the data-driven framework we present here could serve as a model for future systematic refinements in other monogenic hearing loss genes, provided sufficient empirical evidence is available.

Our results also underscore the importance of integrating additional phenotypic and familial data to reduce residual uncertainty. Among the remaining VUS, many were missense variants within functional domains, yet lacked confirmation of IP-III malformation or informative segregation data. Confirmation of IP-III would provide PP4_Strong evidence, potentially upgrading a significant portion of these variants. Similarly, segregation analysis in informative families could contribute PP1_Moderate or PP1_Strong evidence, facilitating further reclassification. These findings highlight that while gene-specific calibration significantly enhances variant classification, its full clinical utility depends on the systematic integration of clinical data, such as radiological evaluations and family-based genetic analysis. Implementing this integrated approach in routine diagnostic workflows can significantly reduce the VUS burden and improve the precision of genetic counselling for families affected by X-linked hearing loss.

The high genotype–phenotype concordance observed between *POU3F4* variants and IP-III malformation (96.4%, 27/28) carries significant clinical implications. Given the strong association between *POU3F4* pathogenic variants and IP-III, we recommend systematic *POU3F4* testing for paediatric patients with IP-III identified on preoperative temporal bone imaging, especially prior to cochlear implantation.^14^ Molecular confirmation of *POU3F4* variants is clinically actionable, as IP-III is associated with abnormal communication between the subarachnoid space and inner ear, predisposing individuals to perilymphatic gushers during surgery.^15^ A preoperative genetic diagnosis can thus guide surgical planning, anticipate cerebrospinal fluid leakage, and optimise perioperative management. Furthermore, identifying an X-linked aetiology allows for better recurrence risk assessment and carrier testing within families. This study demonstrates that gene-specific recalibration not only improves variant classification but also directly informs clinical decision-making, such as surgical risk stratification and personalised management in paediatric cochlear implantation.

In parallel, our systematic evaluation of both upstream and coding CNVs refined the interpretation of noncoding regulatory deletions, highlighting large upstream deletions affecting *POU3F4* as a recurrent pathogenic mechanism in X-linked hearing loss. These deletions underscore the importance of noncoding regulatory variation. When re-evaluating previously reported upstream non-coding deletions under the ACMG/ClinGen 2020 CNV guidelines, some variants did not meet the pathogenic threshold in our re-analysis. This discrepancy is likely due to limited breakpoint resolution in earlier studies, such as MLPA-based reports, where breakpoints were inferred from probe positions.^16^ The more standardised and conservative evidence-weighting system of the 2020 CNV guidelines^17^ may have led to this classification. However, such conservative classification is consistent with clinical relevance, and further refinement of breakpoints, along with segregation data and functional studies, could support future reclassification.

The regulatory interpretation of these CNVs is supported by multiple lines of evidence, including the recurrence of pathogenic deletions in affected individuals and prior functional studies identifying conserved noncoding enhancers in the upstream gene desert.^8^^,^^18^ These findings suggest that disruption of long-range regulatory elements may mimic loss-of-function coding variants by altering *POU3F4* expression during inner ear development. Nonetheless, the precise contribution of individual regulatory elements within large CNVs remains challenging to resolve, highlighting the need for future research that integrates developmental epigenomics and chromatin conformation analyses in relevant cell types.

Additionally, several large deletions in our cohort also affected neighbouring genes such as *CHM*, *TBX22*, and *BRWD3*, which are associated with age-dependent or variably expressive phenotypes. While the systematic evaluation of non-auditory features was limited by patient loss to follow-up or refusal of additional testing, identifying these genes is clinically relevant. In such cases, we aim to provide families with appropriate counselling and information regarding potential future manifestations, as well as recommendations for targeted clinical evaluation when feasible. These considerations emphasise the importance of comprehensive CNV interpretation that goes beyond the primary hearing-loss phenotype, even when overt syndromic features are not immediately present at the time of diagnosis.

Neurodevelopmental abnormalities have been reported in a subset of cases. In our cohort, 5.5% (5/91) probands exhibited neurodevelopmental features, and one carried a multi-gene deletion that may account for the broader phenotype. Clinicians should remain attentive to potential neurodevelopmental concerns and consider referral for appropriate evaluation when indicated.

The treatment of hereditary hearing loss remains an area of active investigation.^19^^,^^20^ In the case of *POU3F4*, stop-loss variants are particularly relevant due to their ability to generate hydrophobic C-terminal extensions that disrupt protein localisation and transcriptional activity. Previous studies have shown that stop-loss variants lead to protein degradation and the accumulation of mislocalised proteins in the cytoplasm. In our study, while the overall protein expression levels of stop-loss variants were lower than WT proteins, rescue experiments involving the addition of a NLS to the N-terminal Myc tag improved nuclear import, even though protein degradation could not be fully prevented. This suggests that restoring nuclear localisation could mitigate some of the pathogenic effects of stop-loss variants, providing a potential therapeutic avenue for these types of mutations.

Interestingly, *POU3F4* also exhibits sex-specific phenotypic variability due to the random and skewed X-inactivation in female carriers. Studies have suggested that females with skewed X-inactivation or escape from inactivation may exhibit progressive hearing loss over time, possibly due to insufficient *POU3F4* expression during ageing. In our cohort, female carriers exhibited variable expressivity, with some displaying normal hearing thresholds and others showing signs of progressive hearing loss, which could be influenced by the dosage of *POU3F4* expression in cochlear cells. We hypothesise that during embryogenesis, minimal expression of *POU3F4* is sufficient for cochlear development, but with age or environmental stressors, higher expression levels are necessary to maintain cochlear function. This insight not only deepens our understanding of progressive hearing loss in female carriers but also suggests a potential therapeutic window for gene therapy targeting *POU3F4*, where increasing its expression could prevent or mitigate the progression of hearing loss in affected individuals.

Despite providing valuable insights, this study has several limitations. While we identified a range of pathogenic variants, the sample size for certain variants, such as missense and structural variants, remains small, which could affect the statistical power of subgroup analyses. Furthermore, while we employed both short-read and long-read sequencing technologies, some structural variants, particularly complex rearrangements or low-frequency variants, may have been missed due to technical limitations in resolution. Finally, although functional assays provided important insights into the impact of stop-loss variants, further in vivo studies are needed to fully understand the long-term consequences and the role of potential genetic modifiers.

This study provides a gene-specific refinement of ACMG/AMP evidence for *POU3F4*-related hearing loss, integrating large-scale cohort data, paralog-conservation analysis, and functional validation. By redefining evidence categories, resolving subsets of VUS, and elucidating pathogenic mechanisms of structural and stop-loss variants, our work establishes a replicable framework for gene-specific variant interpretation. These advances address a critical unmet need in clinical genetics and offer a model for precision diagnosis of monogenic disorders.

## Contributors

J.G., Yix.Z., and Y.H. contributed equally to this work. J.G., Yix.Z., Y.H., F.B., J.C., Y.L., and H.Y. conceived and designed the study. J.G., Y.H., W.X., C.W., X.W., T.T., M.C., L.F., and Y.Y. performed experiments, including genetic sequencing, variant analysis, and functional assays. J.G., Yix.Z., Y.H., M.Z., and Q.Z. analysed the data and performed statistical analyses. J.G., Yix.Z., and Y.H. draughted the manuscript. F.B., J.C., Y.L., and H.Y. critically revised the manuscript for important intellectual content and supervised the study. J.G., Yix.Z., Y.H., F.B., J.C., Y.L., and H.Y. had full access to the data and verified the underlying data. All authors reviewed and approved the final version of the manuscript.

## Data sharing statement

The sequencing and clinical data included in this study are not publicly available due to privacy and legal issues. Controlled data access can be applied to the corresponding author (yuanhj301@wchscu.cn) upon reasonable request with IRB approval. Please allow up to three months from request to data sharing for regulatory compliance. All methods and analytical procedures are described in sufficient detail in the manuscript and Supplemental Methods to allow reproducibility.

## Declaration of interests

All the authors declare no competing interests.

## Appendix A Supplementary data

Supplemental Tables

Reagent validation

Full unedited gel
