# Supplementary material for: Optimising POU3F4 variant interpretation through gene-specific evidence in X-linked hearing loss
Source: eBioMedicine. 2026 May 29;128:106318. doi: 10.1016/j.ebiom.2026.106318 (PMC13242011; doi:10.1016/j.ebiom.2026.106318)
Supplement: Supplemental Figures [file mmc1.pdf]

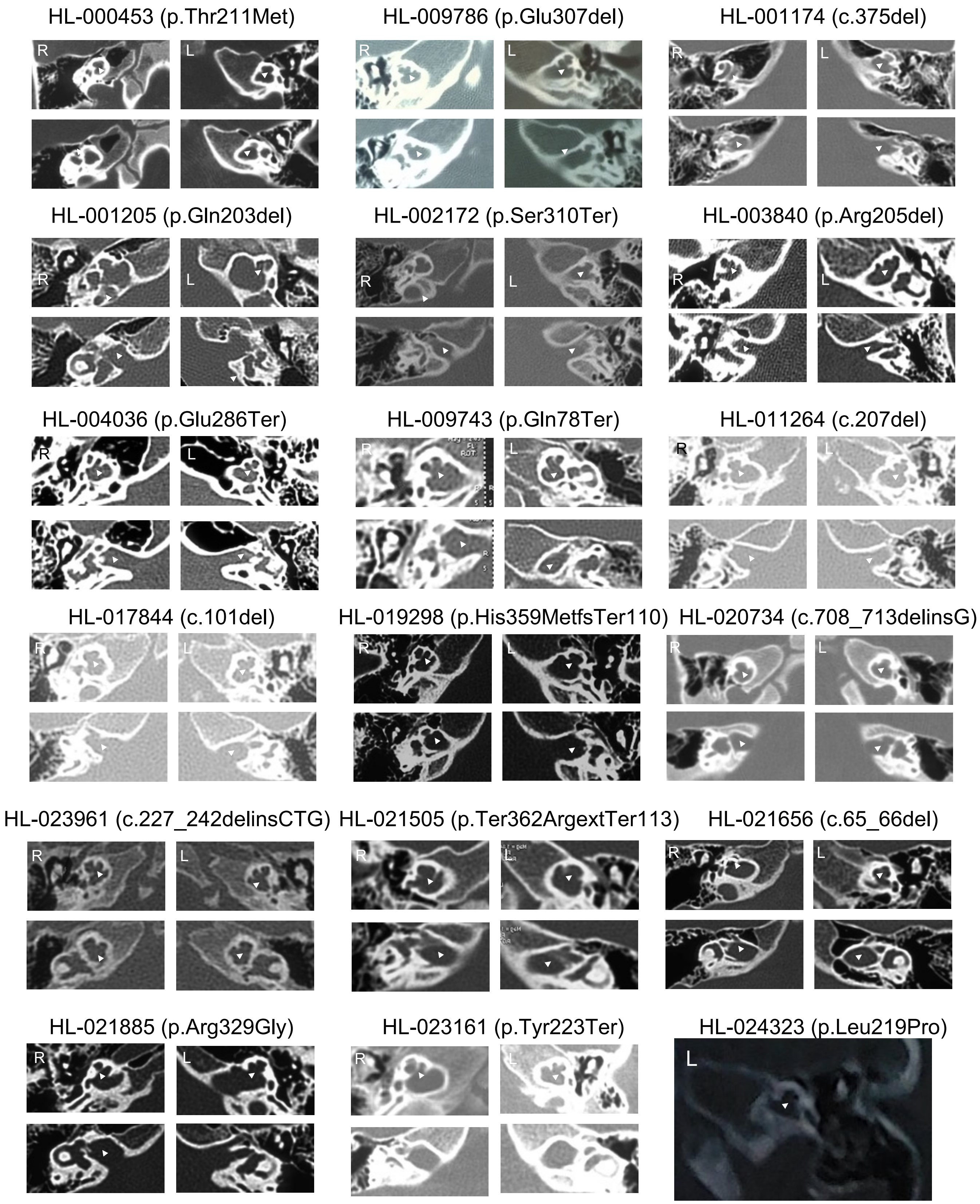

Supplemental Figure 1. Temporal bone computerized tomography (CT) on patients with disease-causing *POU3F4* variants. All CTs performed IP III malformation with absent of modiolus (arrow in upper picture) and enlarged internal auditory canal (arrow in lower picture).

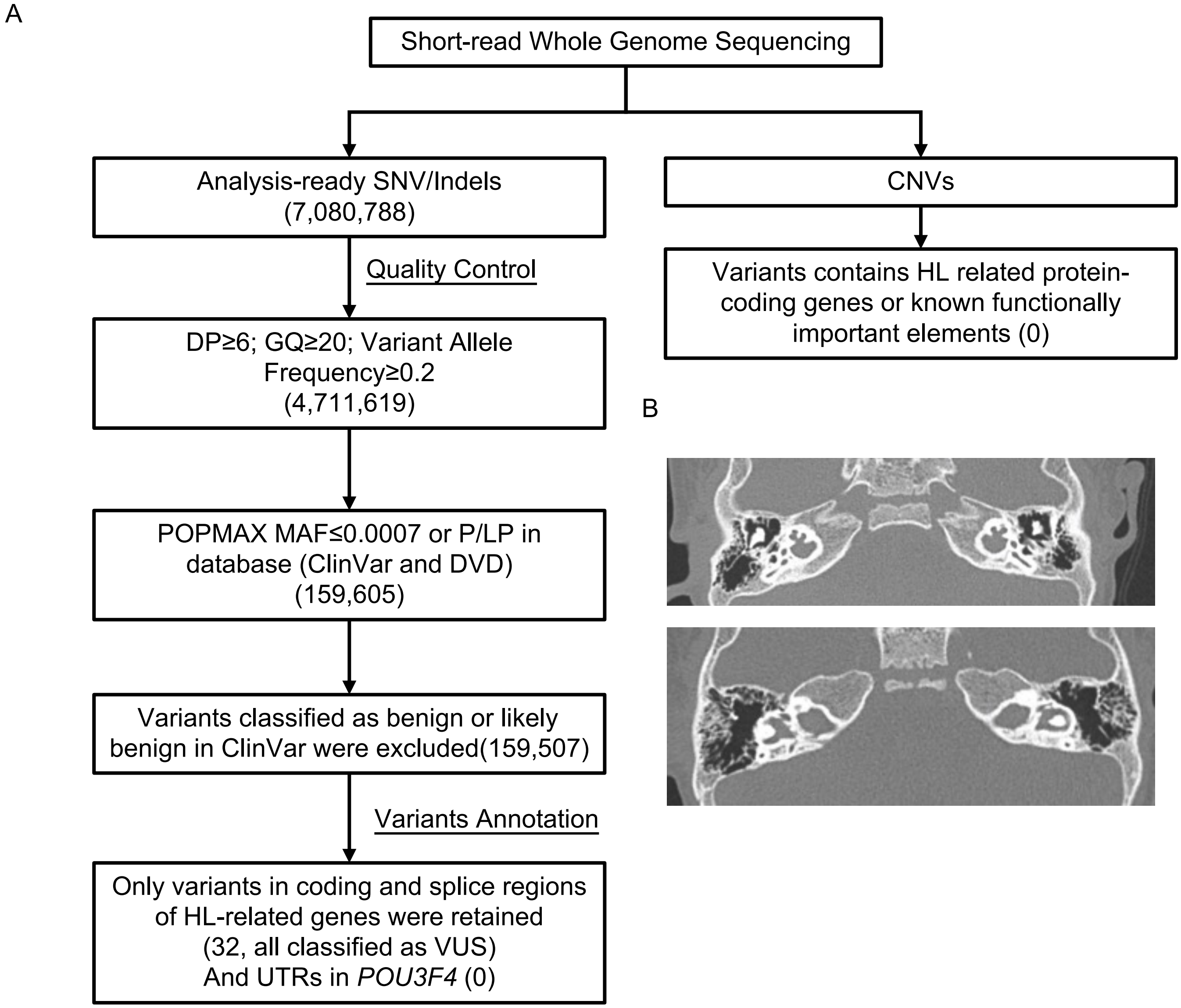

Supplemental Figure 2. Temporal bone CT and variants analysis workflow of the undiagnosed female patient with IP-III malformation. (A) Analysis-ready variants underwent quality control, followed by population frequency filtering and the exclusion of benign or likely benign variants. The final set includes variants in coding or splice regions of hearing-loss-related genes, all classified as variants of uncertain significance. No variants were identified in untranslated regions of the *POU3F4* gene. (B) Temporal bone CT showed absence of the modiolus and enlargement of the internal auditory canal, consistent with the classic findings of IP-III malformation. SNV/Indels, single nucleotide variants and Insertion/deletions; DP, depth of coverage; GQ, genotype quality; VUS, variant of uncertain significance; HL, hearing loss; MAF, minor allele frequency.

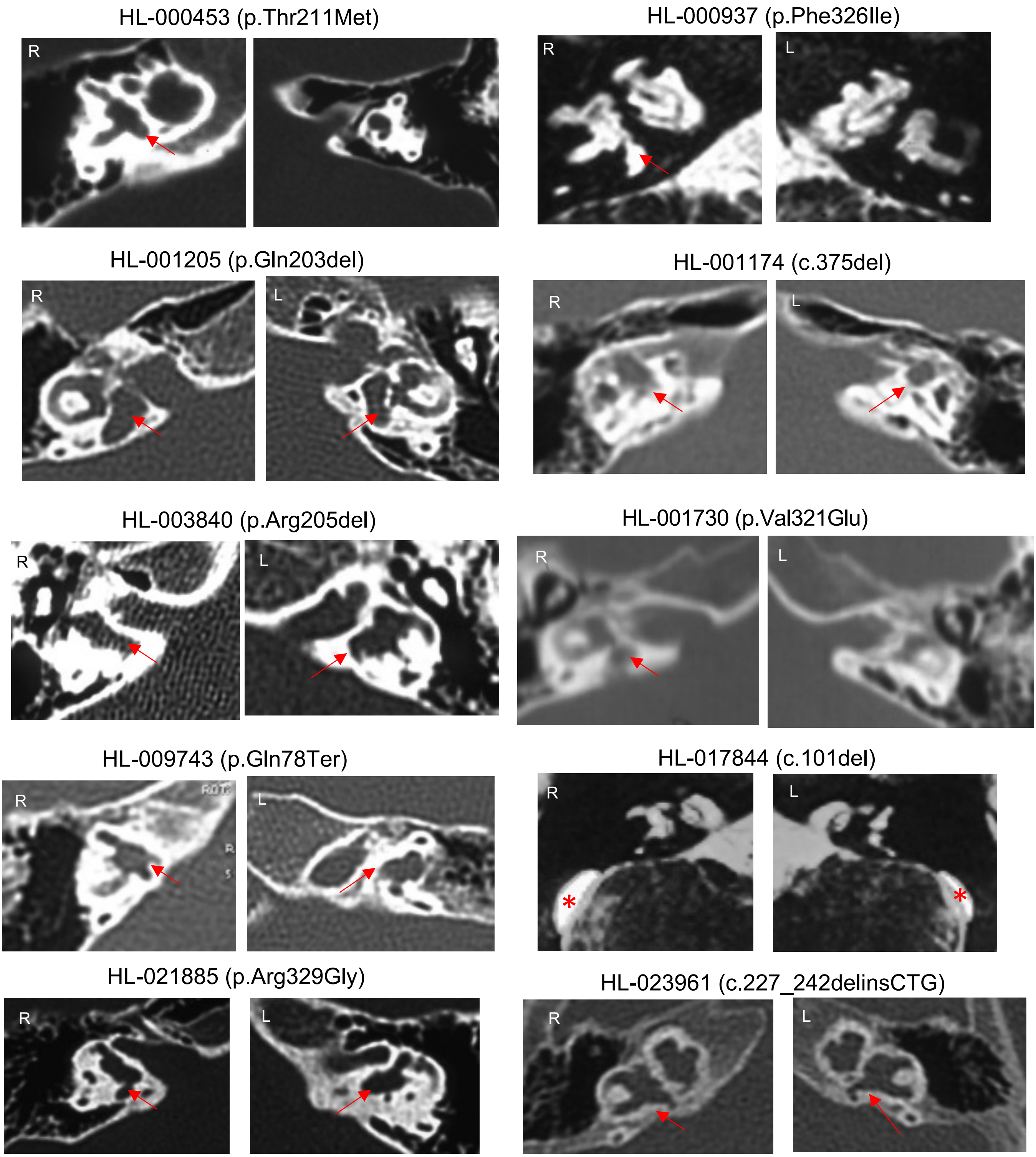

Supplemental Figure 3. Radiologic features of enlarged vestibular aqueduct (EVA) in *POU3F4*-related IP-III patients. Representative temporal bone CT or MRI images from 10 patients are shown. Red arrows indicate enlargement of the vestibular aqueduct, predominantly involving the mid-portion and cochlear end. Asterisks (*) on MRI images denote enlargement of the endolymphatic sac secondary to EVA.

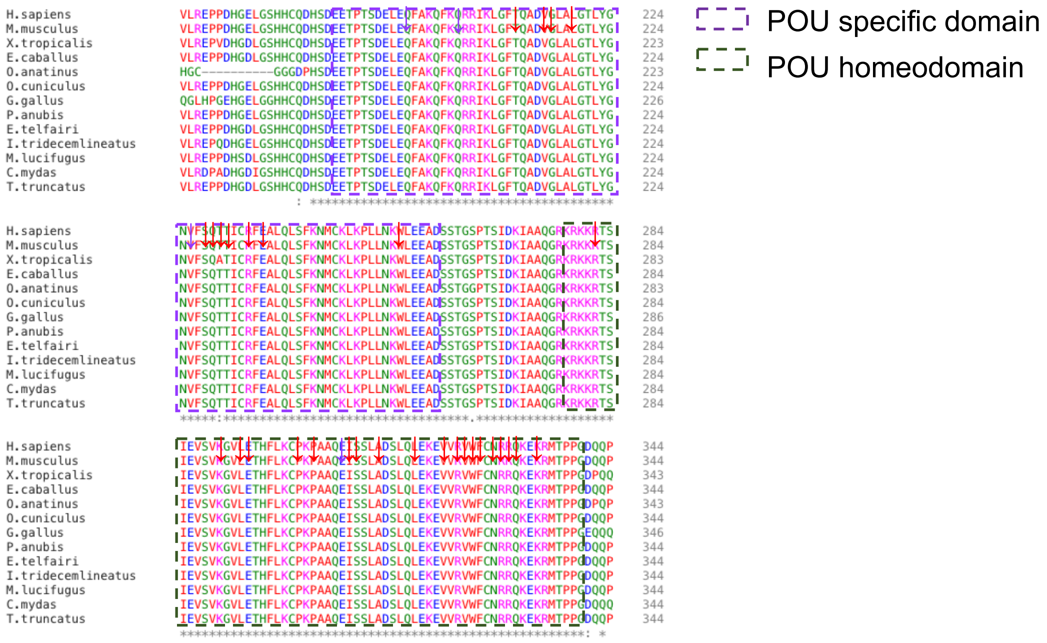

Supplemental Figure 4. Homology conservation of POU3F4 protein in difference species. Alignment of the POU domains sequence of vertebrate POU3F4 orthologs, with indication of residues altered in HL patients (red) and altered gnomAD male individuals (purple). Asterisks, fully conserved residues. Dots, semi-conserved residues.

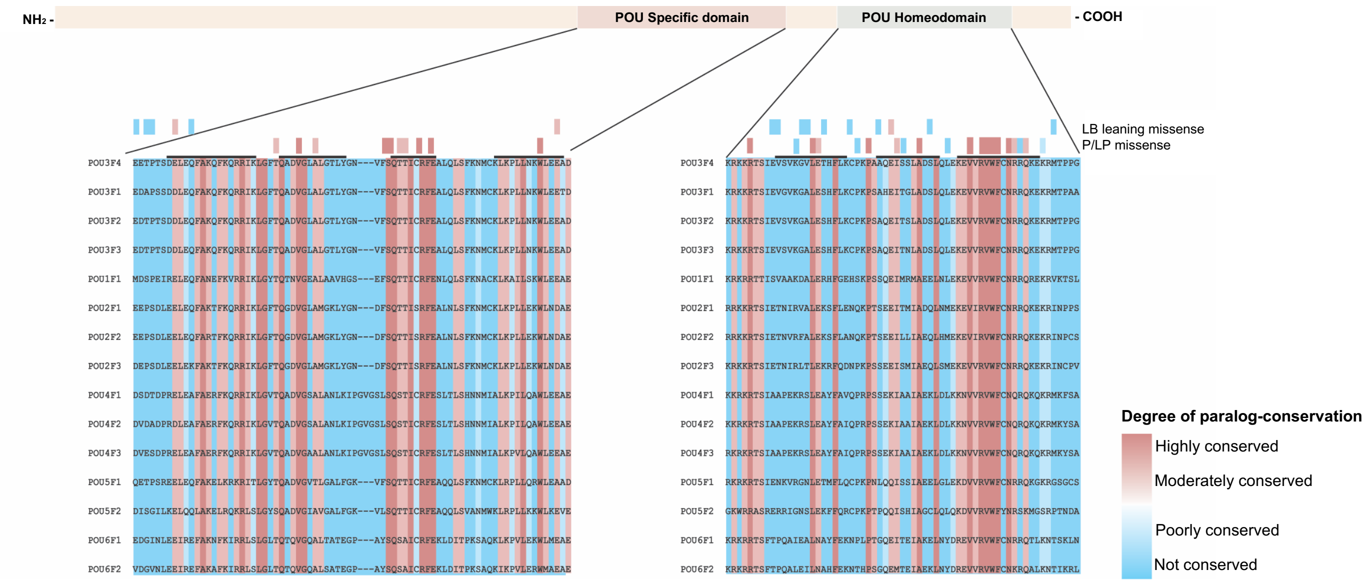

Supplementary Figure 5. Full multiple-sequence alignment of POU3F4 POU-specific domain and POU-homeodomain across POU-family paralogs.

To support the conservation analysis summarized in **Figure 2C**, we provide the complete paralogous multiple-sequence alignment for the two DNA-binding domains of POU3F4 (POU-specific domain and POU-homeodomain) across POU-family proteins. Domain structure of the POU3F4 protein, highlighting the POU-specific domain (pink) and homeodomain (green). LB leaning missense (hemizygous missense variants from gnomAD v4.1.0) are shown above the schematic, while P/LP missense variants are shown below. Multiple sequence alignment of the two domains from 15 POU family proteins is displayed in the lower panel, with paralog conservation indicated by a color scale (red = high, blue = low).

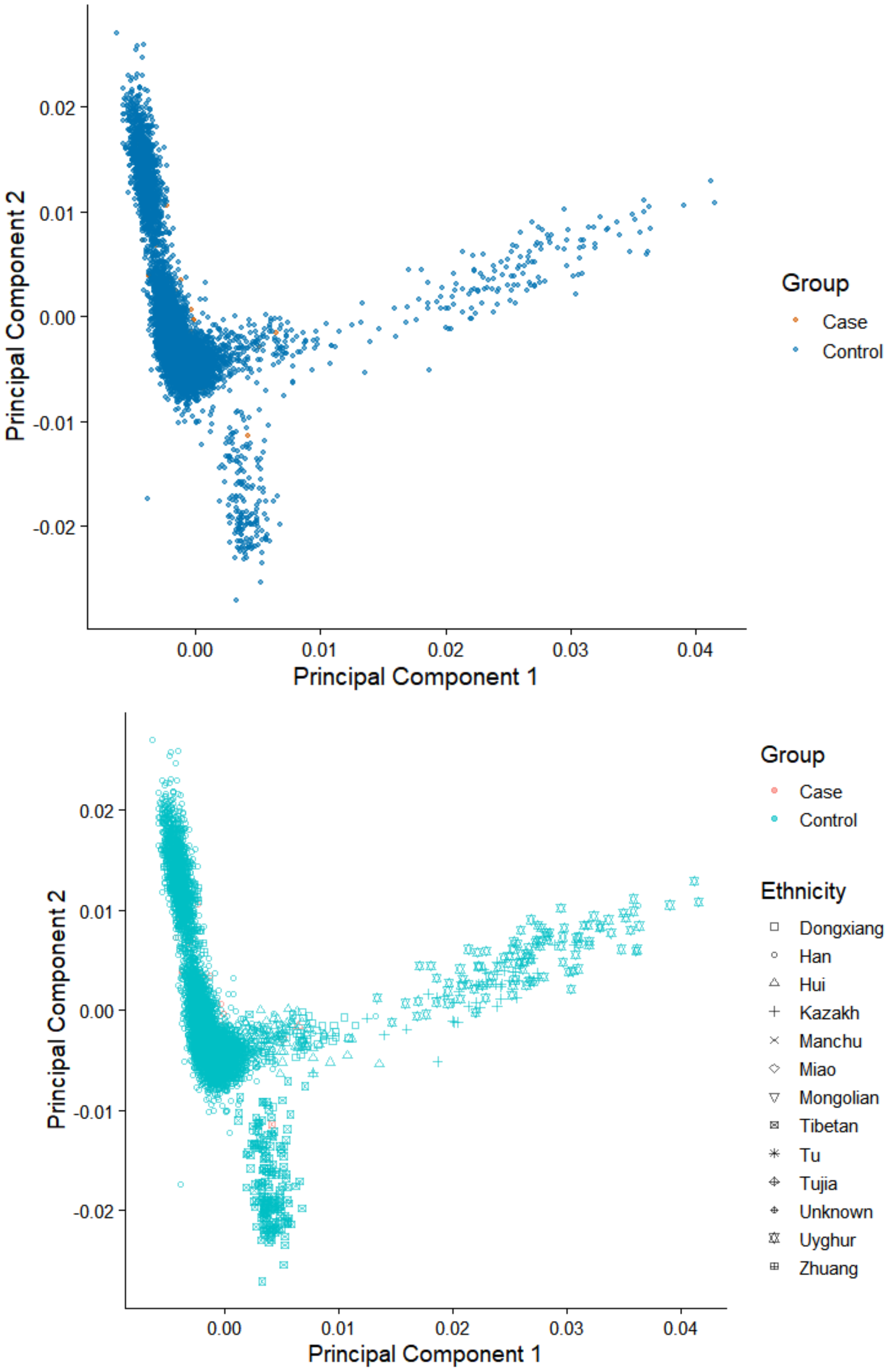

Supplementary Figure 6. Principal component analysis (PCA) of cases and controls.
Upper panel: Scatter plot of the first two principal components (PC1 and PC2) for 20,666 unrelated hearing loss cases and 7,258 controls, colored by case/control status. No evident clustering or separation between groups was observed.
Lower panel: PCA plot stratified by self-reported ethnicity, showing expected population structure with cases and controls well mixed within clusters.

HL-012890 NC_000023.10:g.80551315-85061186del

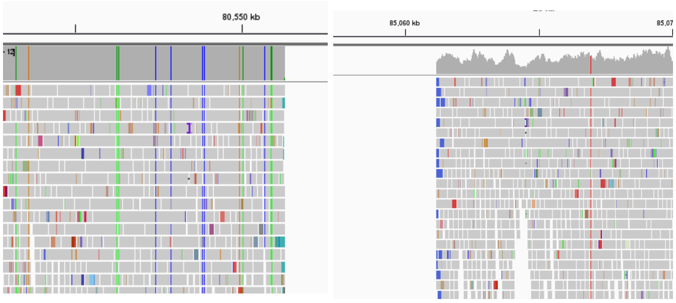

HL-008079 NC_000023.10:g.81299432-83512874del

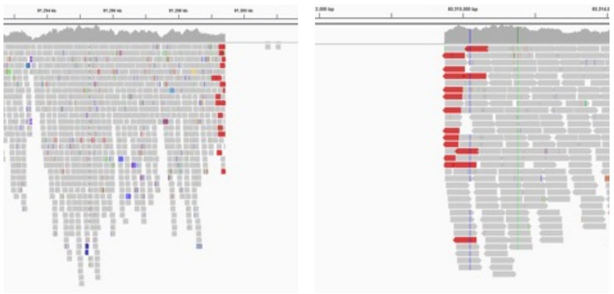

HL-013285 NC_000023.10:g.81841279-89668337del

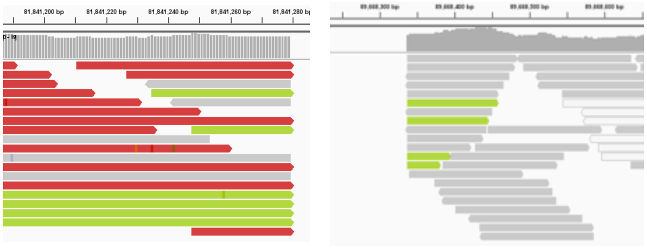

HL-000577 NC_000023.10:g.82043507-82854212del

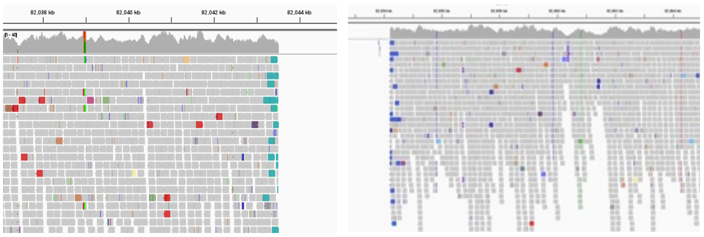

HL-004917 NC_000023.10:g.82267902-82771732del

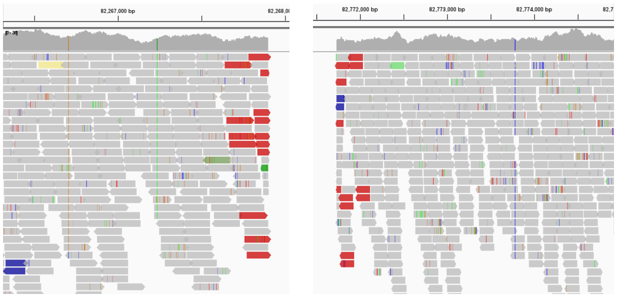

HL-004105 NC_000023.10:g.82284714-89391869del

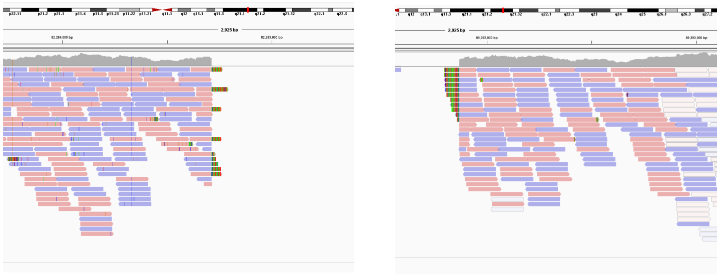

HL-017877 NC_000023.10:g.82318260-85373609del

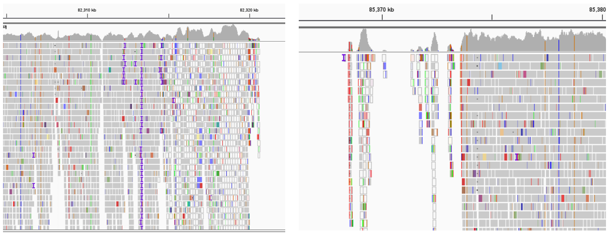

HL-013575 NC_000023.10:g.82364967-84226187del

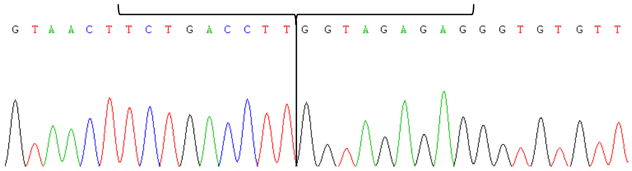

HL-007687 NC_000023.10:g.82473841-83908624del

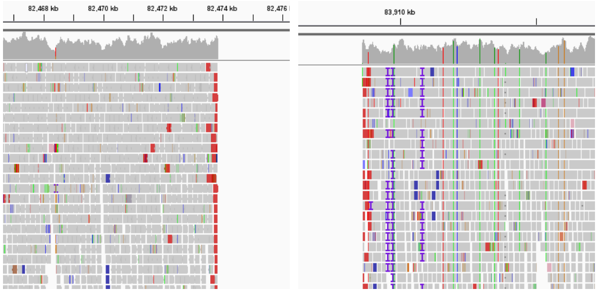

HL-000714 NC_000023.10:g.82398115-92930582del

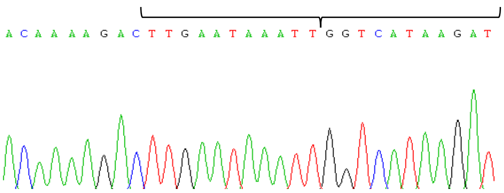

HL-041599 NC_000023.10:g.82521444-82966045del

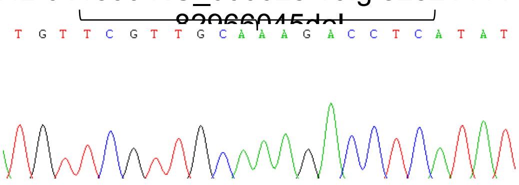

HL-009371 NC_000023.10:g.82639387-89968789del

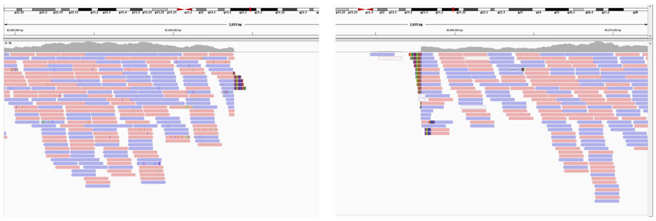

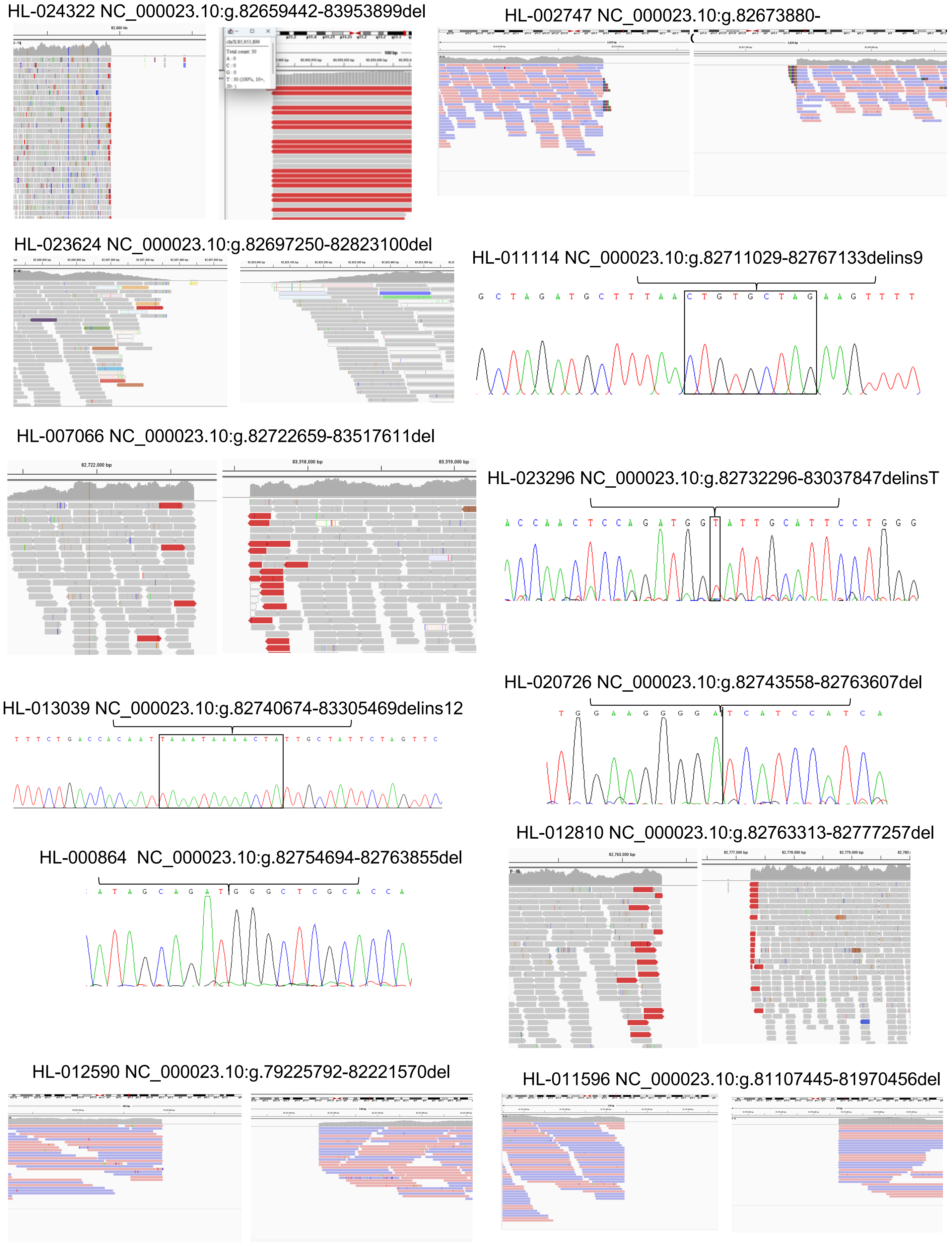
HL-024322 NC_000023.10:g.82659442-83953899del
HL-002747 NC_000023.10:g.82673880-
HL-023624 NC_000023.10:g.82697250-82823100del
HL-011114 NC_000023.10:g.82711029-82767133delins9
HL-007066 NC_000023.10:g.82722659-83517611del
HL-023296 NC_000023.10:g.82732296-83037847delinsT
HL-020726 NC_000023.10:g.82743558-82763607del
HL-013039 NC_000023.10:g.82740674-83305469delins12
HL-012810 NC_000023.10:g.82763313-82777257del
HL-000864 NC_000023.10:g.82754694-82763855del
HL-012590 NC_000023.10:g.79225792-82221570del
HL-011596 NC_000023.10:g.81107445-81970456del

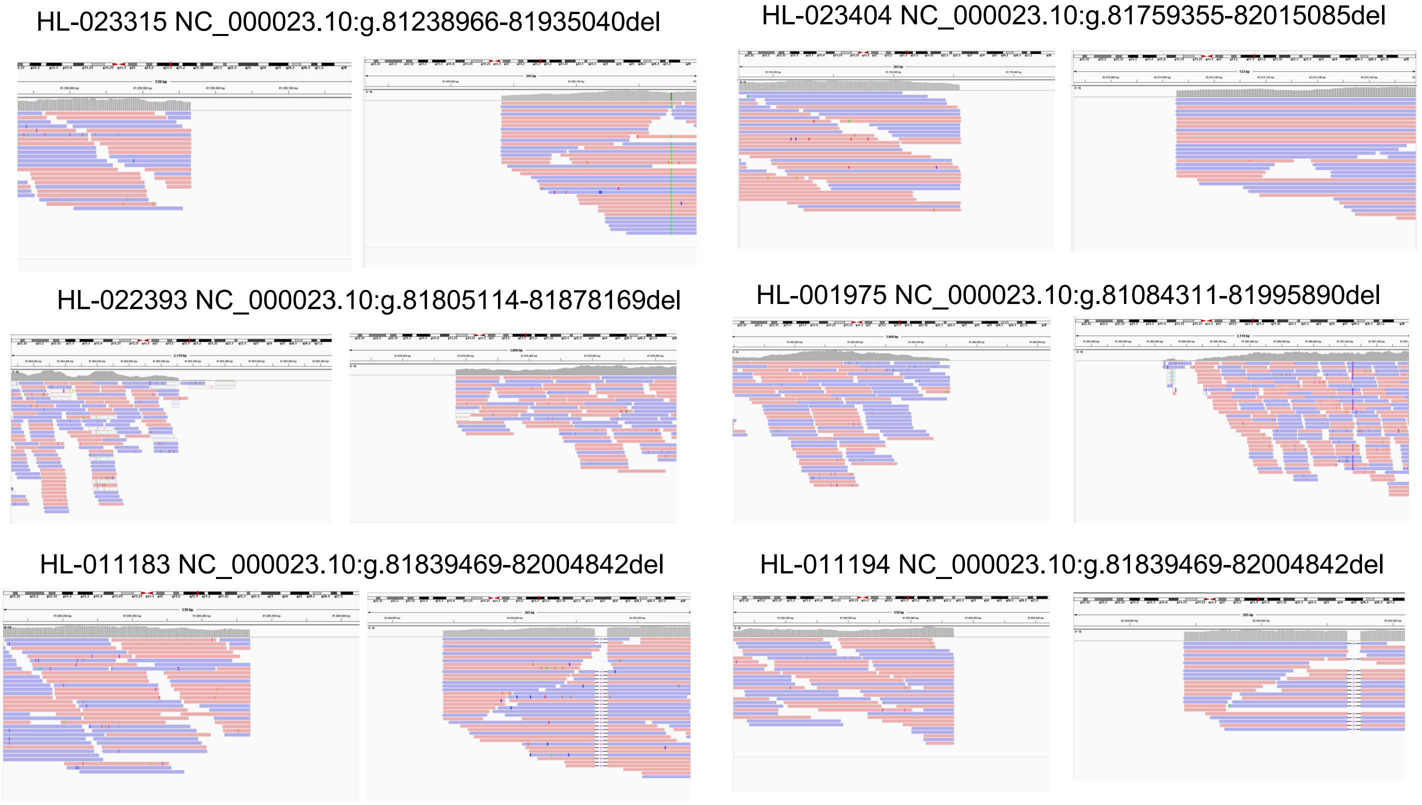

Supplemental Figure 7. IGV_2.4.4 visualization or Sanger sequencing validation of *POU3F4* gene related deletion regions identified in CDGC cohort. Reference genome: GRCh37.

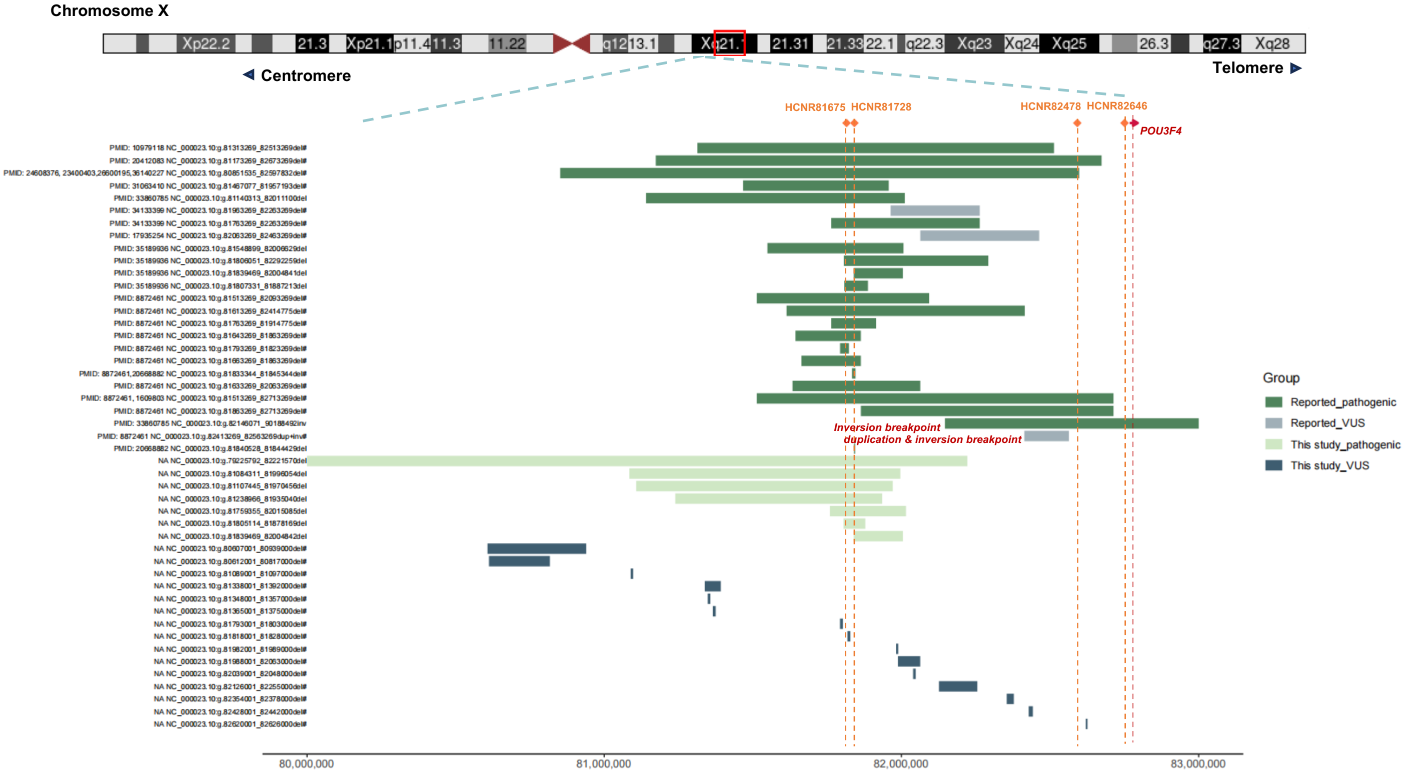

Supplemental Figure 8. Schematic representation of large genomic deletions located upstream of the *POU3F4* gene on chromosome X. The ideogram at the top indicates the position of the analyzed region (Xq21.1) relative to the centromere and telomere. Seven deletions identified in eight patients in this study are shown, all of which are located upstream of *POU3F4* and overlap regions containing known enhancer elements. Previously reported pathogenic deletions and variants of uncertain significance (VUS) are shown in green and gray, respectively, whereas deletions identified in this study are shown in light green (pathogenic) and dark blue (VUS). Red triangles denote previously characterized highly conserved noncoding regions (HNCRs), which have been functionally validated in prior studies as enhancers regulating *POU3F4* expression during inner ear development. Orange dashed lines indicate reported inversion and duplication/inversion breakpoints within the region. Genomic coordinates are shown along the x-axis.

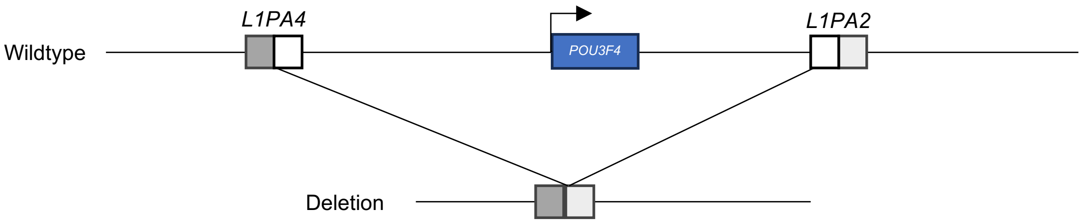


```
L1PA4      AAGTGGGCAAAGGATATGAACAGACACTTCTAGAAAGAAGACATTTATGCAGCCAAGAGA 60
L1PA2      AAGTGGGTGAAGGACATGAACAGACACTTCTCAAAAGAAGACATTTATGCAGCCAAAAAA 60
           *******  ***** ****************  *********************** * *

L1PA4      CACATGAAAAAATGTTCATCATCACTGACCATCAGAGAAATGCAAATCAAAACCACTATG 120
L1PA2      CACATGAAAAAATGCTCATCATCACTAGCCATCAGAGAAATGCAAATCAAAACCACTATG 120
           ************** ***********  ********************************

L1PA4      AGATACCATCTCACACCAGTTAGAATGGCAATCATTAAAAAGTCAGGAAACAACAAGTGC 180
L1PA2      AGATACCATCTCACACCAGTTAGAATGGCAATCATTAAAAAGTCAGGAAACAACAGCTGC 180
           *******************************************************  ***

L1PA4      TGGAGAGGATATGGAGAAAT 200
L1PA2      TGGAGAGGATGTGGAGAAAT 200
           ********** *********
```

Supplementary Figure 9. Repeat-rich breakpoint context and sequence similarity for a LINE-1–associated deletion at the *POU3F4* locus.
Schematic (top) illustrates the genomic organization of the deletion, in which the two breakpoints map within LINE-1 elements belonging to different subfamilies (L1PA4 and L1PA2) flanking the *POU3F4* region. Alignment (bottom) shows 200-bp sequences extracted from the breakpoint-flanking regions of L1PA4 and L1PA2; asterisks indicate identical nucleotides. The presence of extensive local sequence similarity supports that this specific deletion may have arisen within a repeat-rich genomic context.

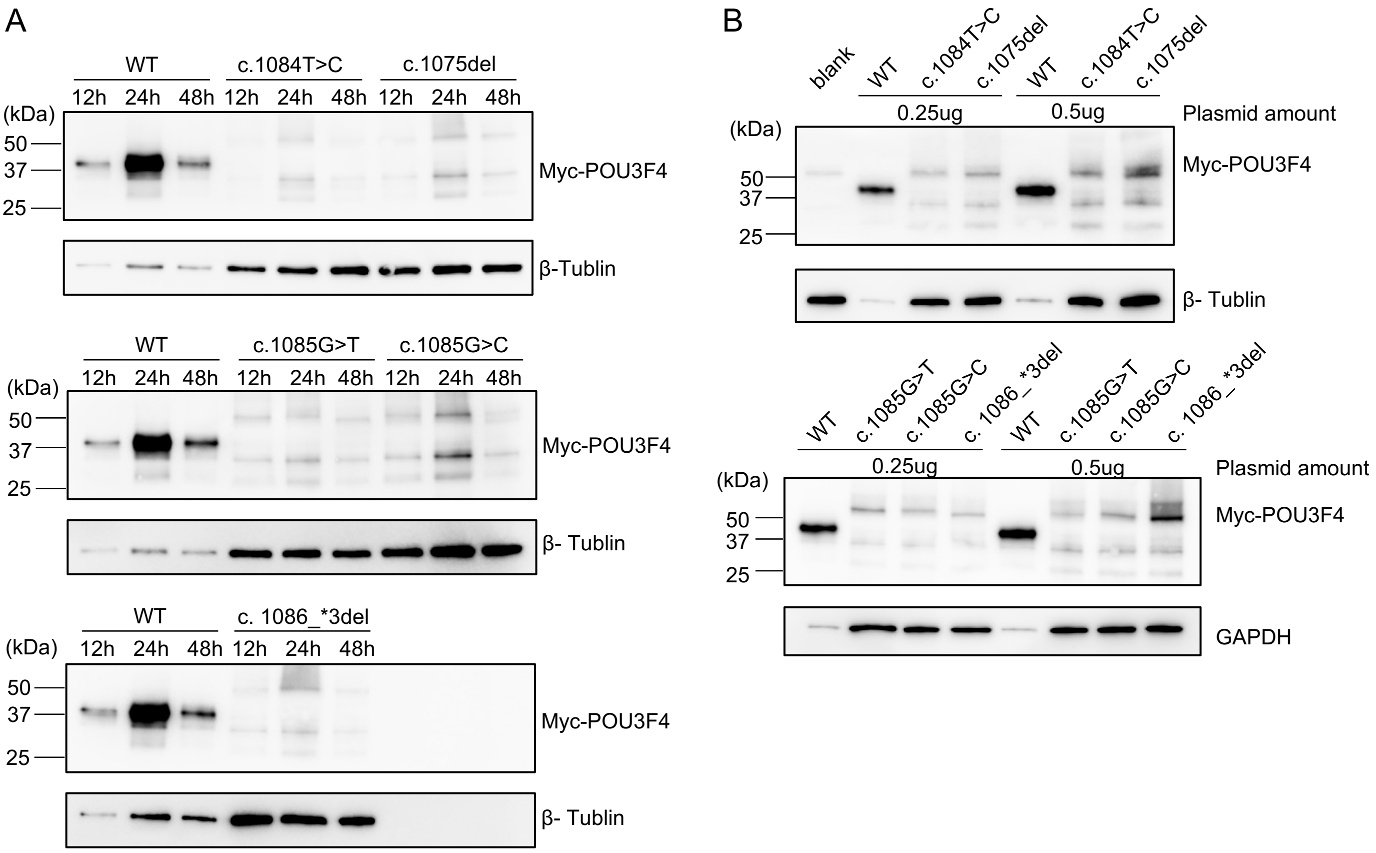

Supplemental Figure 10. Western blot result. A. Temporal expression profile of POU3F4 WT and stop-loss variants. HEK293T cells were transfected with Myc-tagged WT or stop-loss variants (500 ng) and harvested at 12 h, 24 h, and 48 h post-transfection. WT samples were included in each blot for direct comparison. β-tubulin served as loading control. Molecular weight markers (50, 37, and 25 kDa) are indicated. Variants are labeled according to HGVS cDNA nomenclature. **B.** Western blot analysis of Myc-tagged WT and stop-loss variants transfected at 250 ng or 500 ng and harvested at 24 h post-transfection. Blank vector was included where indicated. β-tubulin or GAPDH were used as loading control. Molecular weight markers (50, 37, and 25 kDa) are shown.

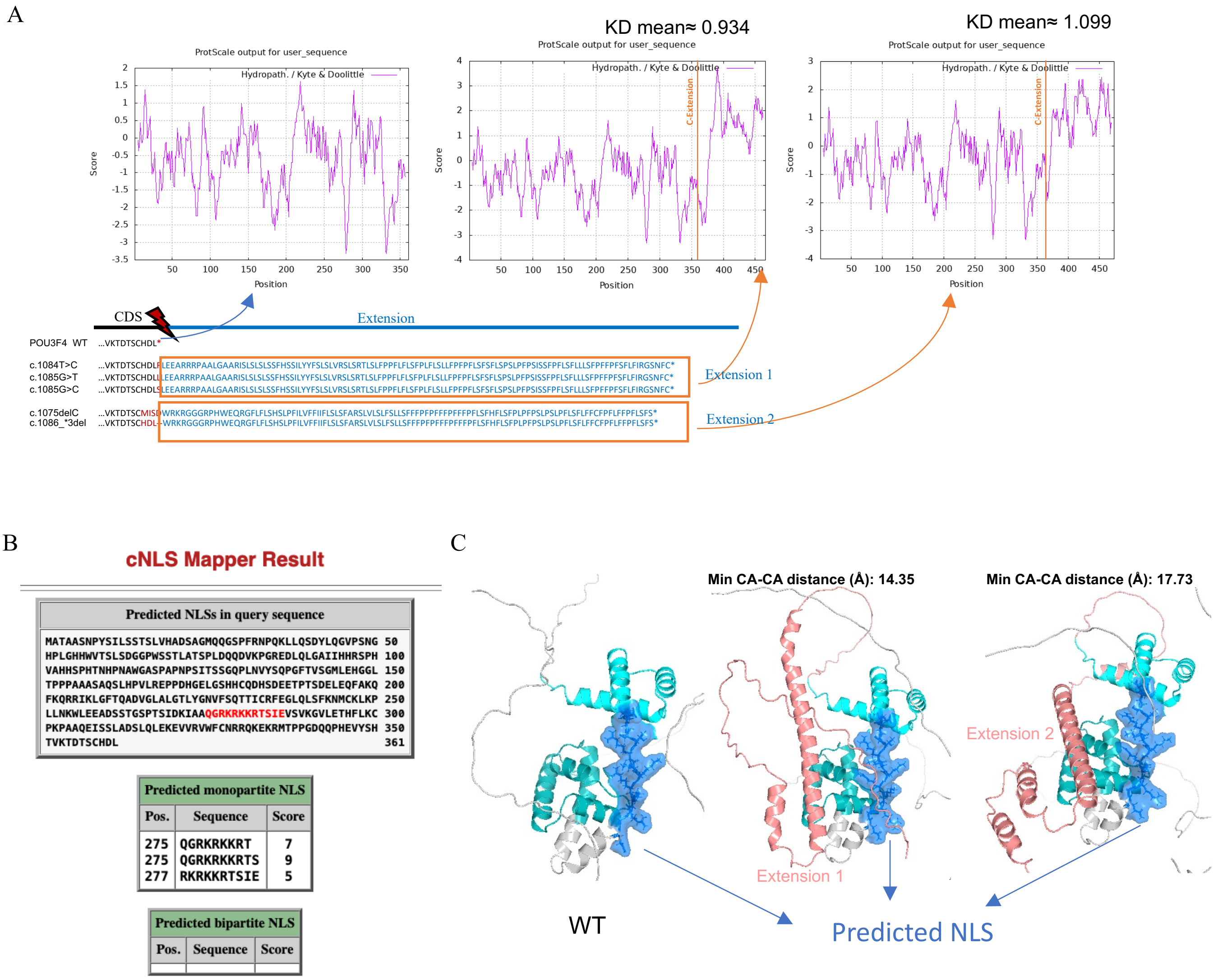

Supplemental Figure 11. Structural studies of stop-loss variants. A. Hydropathy plot generated using Kyte and Doolittle scale shows the presence of a long stretch of hydrophobic amino acids in the extended C-terminal region demarcated by yellow line otherwise absent in the wild-type protein. **B**. nuclear localization signal sequence predicted by NLS Mapper (https://nls-mapper.iab.keio.ac.jp/cgi-bin/NLS_Mapper_form.cgi). **C**. Structural modeling of wild-type (WT) POU3F4 and two representative stop-loss extension variants generated using AlphaFold2. The POU-specific domain and POU homeodomain are shown in cyan. The predicted nuclear localization signal (NLS; residues 275–286) is highlighted in dark blue. C-terminal extension peptides are shown in red. Structural models indicate spatial proximity between the extension peptides and the predicted NLS, with minimum Cα–Cα distances of 14.35 Å (Extension 1) and 17.73 Å (Extension 2), supporting a potential conformational interference mechanism affecting nuclear import.
