# Supplementary material for: Optimising POU3F4 variant interpretation through gene-specific evidence in X-linked hearing loss
Source: eBioMedicine. 2026 May 29;128:106318. doi: 10.1016/j.ebiom.2026.106318 (PMC13242011; doi:10.1016/j.ebiom.2026.106318)
Supplement: Supplemental Appendix [file mmc2.docx]

**Supplementary Appendix**

**Supplementary Methods**

**Cohort Recruitment and** **Demographics**

The Chinese Deafness Genetics Consortium (CDGC) is a nationwide collaborative project established in September 2013 to investigate the genetic basis of hearing loss (HL) across mainland China. Between September 2013 and February 2022, a total of 22,474 individuals with hearing impairment were recruited from 91 special education schools, 89 rehabilitation centers for deaf children, and 34 tertiary hospitals spanning all 31 provincial-level administrative regions of mainland China.

Participants underwent standardized clinical evaluation, including review of medical history, physical examination, and pure-tone audiometry. Individuals were excluded if hearing impairment was attributable to conductive causes (n = 9), presbycusis (n = 150), unilateral hearing loss (n = 57), or mild hearing impairment (pure-tone average ≤40 dB; n = 133). After exclusion, 22,125 individuals from 3,662 multiplex families and 17,003 simplex families remained eligible.

For genetic association analyses, only unrelated individuals were included to avoid familial overrepresentation. After removal of related individuals, 20,666 unrelated cases (hereafter referred to as “cases”) were retained for downstream analyses.

The cohort predominantly consisted of individuals with early-onset hearing loss (onset <7 years, 96.0%), severe-to-profound impairment (>70 dB, 94.7%), and nonsyndromic presentation (98.8%). The geographic distribution of participants covered 314 of 333 prefectural-level regions in China and was consistent with national population distribution patterns. Regarding ethnicity, Han Chinese constituted the majority of cases (17,028 individuals, 82.4%), while 3,334 individuals (16.1%) belonged to 40 minority ethnic groups. Ethnicity information was self-reported.

The control group comprised 7,258 unrelated adults (>18 years) without self-reported hearing loss, recruited through the CDGC consortium and the Fudan Huabiao project.

To evaluate potential population stratification between cases and controls, principal component analysis (PCA) was performed based on common variants derived from genome-wide sequencing data. The first two principal components showed substantial overlap between cases and controls, with no apparent clustering by case–control status. Similarly, stratification by major ethnic groups did not reveal distinct separation between cases and controls, supporting minimal population structure bias in downstream analyses.

The study protocol was approved by the ethics committees of West China Hospital and Southwest Hospital. Written informed consent was obtained from all participants or their legal guardians in accordance with the Declaration of Helsinki.

**DNA Extraction and Sequencing**

Peripheral blood samples were collected from all participants and available family members. Genomic DNA was extracted using the MagNA PURE 96 system (Roche, Germany) according to the manufacturer’s protocol. DNA quality and concentration were assessed by spectrophotometry and agarose gel electrophoresis prior to library preparation.

Initial variant screening was performed using a multiplex SNPscan assay (Shanghai Genesky Biotech, Shanghai, China), covering 96 SNVs, 19 insertions/deletions (indels), and 3 CNV loci in *GJB2*, *SLC26A4*, and *MT-RNR1*.

Genetically undiagnosed patients and controls underwent 157 hearing loss–related genes using the CDGC-HL capture panel (Agilent SureSelect Target Enrichment System). Exonic regions and ±25 bp of flanking intronic sequences were captured. Libraries were prepared according to standard protocols and sequenced on an Illumina platform with paired-end reads.

Genetically undiagnosed patients after targeted panel testing underwent short-read genome sequencing (GS) using the DNBSEQ-T7 platform (BGI, Shenzhen, China) with 150 bp paired-end reads.

Sequencing reads were aligned to the human reference genome (GRCh37/hg19) using Burrows–Wheeler Aligner (BWA). Variant calling was performed following Genome Analysis Toolkit (GATK) best practices. Variants were annotated using Ensembl Variant Effect Predictor (VEP). Copy number variants (CVs) were detected using CNVnator and Manta. Non-coding CNVs located upstream of *POU3F4* were annotated for overlap with candidate cis-regulatory elements using publicly available databases, including ENCODE4 Registry of candidate cis-regulatory elements, SEdb, HEDD, HACER, GREENDB, FANTOM, and DiseaseEnhancer.

For one female individual with radiologically confirmed IP-III but no candidate variants identified by panel or short-read genome sequencing, long-read sequencing was performed using Oxford Nanopore Technologies (ONT). Genomic DNA was isolated using the SolPure Blood DNA Kit I (Magen, D3311). Libraries were prepared using the Ligation Sequencing Kit V14 (SQK-LSK114; Oxford Nanopore Technologies) and sequenced on a PromethION platform for 72 hours using an R10.4.1 flow cell Basecalling was performed using Dorado (v7.4.12) within MinKNOW (v24.06.10). Reads were aligned to the T2T-CHM13 v2.0 reference genome using Minimap2 (v2.30). Alignments were sorted and indexed with SAMtools (v1.12). Structural variants (>50 bp, including deletions, duplications, inversions, and translocations) were jointly called using Delly (v1.1.3) and Sniffles2 (v2.0.7).

**Variant Annotation and Filtering Criteria**

Single-nucleotide variants (SNVs) and small insertions/deletions (indels) were annotated using Ensembl Variant Effect Predictor (VEP). Only variants meeting the following quality control criteria were retained for analysis: sequencing depth (DP) >5 and genotype quality consistent with hemizygous state in male individuals.

To enrich for rare potentially pathogenic variants, variants with minor allele frequency (MAF) >0.01 in gnomAD (v4.1.0) or the in-house CDGC control dataset were excluded. For hemizygous X-linked variants, any variant observed in hemizygous males in gnomAD was excluded from pathogenic consideration, given the established high penetrance of *POU3F4*-related IP-III malformation. Synonymous variants were excluded unless predicted to affect splicing. Missense variants were further evaluated using multiple in silico prediction tools, including CADD, MCAP, PolyPhen-2, SIFT, GERP++, phyloP, and AlphaMissense. These predictions were used as supportive information but were not solely relied upon for final classification.

CNVs spanned the *POU3F4* coding region or extended up to 1 Mb upstream were retained. Filtering criteria included allele frequency <1% in public databases (gnomAD, 1000 Genomes), minimal overlap with known benign variants in the Database of Genomic Variants (DGV), overlap <25% with segmental duplications or repeat low-complexity regions (RLCRs), detected in fewer than 20 individuals within the CDGC cohort, and read-depth thresholds between 10× and 300×.CNVs were interpreted according to the 2020 ClinGen CNV interpretation guidelines. All candidate CNVs were manually reviewed using Integrative Genomics Viewer (IGV, v2.4.10). Breakpoints were refined by Sanger sequencing or BAM file inspection when possible.

In addition to genotype-driven filtering, individuals with radiologically confirmed incomplete partition type III (IP-III) malformation identified by temporal bone CT imaging were screened for *POU3F4* variants regardless of initial filtering results. IP-III diagnosis was established by experienced otologists based on characteristic findings, including absence of the modiolus and widening of the internal auditory canal.

**Functional Assays and Structural Modeling**

The full-length open reading frame (ORF) of *POU3F4* was PCR-amplified from genomic DNA obtained from an adult male control individual without hearing loss in the CDGC cohort. The amplified fragment, including part of the 3′ untranslated region, was subcloned into the pCMV-Myc-N vector (Cat No. P0816, MiaoLingBio, China), generating an N-terminal Myc-tagged expression construct.

Stop-loss variants were introduced by site-directed mutagenesis using the Fast Site-Directed Mutagenesis Kit (Cat. No. 4992901, TIANGEN, China) according to the manufacturer’s protocol. For rescue experiments, an SV40 nuclear localization signal (NLS; PKKKRKV) was inserted immediately upstream of the Myc tag in each mutant construct, generating corresponding NLS-POU3F4 rescue plasmids. All constructs were verified by Sanger sequencing. All constructs were verified by Sanger sequencing. HEK293T cells (RRID: CVCL_0063; Chinese Academy of Sciences Cell Bank, Cat. No. GNHu17) and HeLa cells (RRID: CVCL_0030; Chinese Academy of Sciences Cell Bank, Cat. No. TCHu187) were used in this study. HEK293T and HeLa cells were maintained in DMEM supplemented with 10% fetal bovine serum and 1% penicillin–streptomycin at 37°C with 5% CO₂. Cells were transfected using Lipofectamine 3000 (Cat. No. L3000015, Thermo Fisher Scientific) according to the manufacturer’s instructions. Plasmid DNA was transfected at 250 ng or 500 ng as indicated.

For western blot assay, HEK293T cells were harvested for protein extraction at 12 h, 24 h, or 48 h post-transfection. Total cellular protein was extracted using RIPA buffer supplemented with protease inhibitors. Protein concentrations were determined using a BCA assay. Equal amounts of total protein were separated by SDS-PAGE and transferred onto PVDF membranes. For wildtype (WT) samples, 3 μg total protein was loaded per lane. For stop-loss variant and rescue constructs, 20 μg total protein was loaded per lane. Membranes were incubated with Anti-c-Myc primary antibody (1:5000, Cat. No. M4439, Sigma) followed by HRP-conjugated secondary antibody (1:1000, Cat. No. A0216, Beyotime) and chemiluminescent detection. After imaging, membranes were stripped and reprobed with anti-β-Tubulin (1:2000, Cat. No. 802001, BioLegend) or anti-GAPDH(1:2000, Cat. No. D190090-0100, Sangon Biotech) as loading controls.

For immunocytochemistry, HeLa cells harvested at 24h were fixed with 4% paraformaldehyde, permeabilized with 0·5% Triton, and incubated with anti-c-Myc antibody (1:2000, Cat. No. M4439, Sigma) followed by Alexa Fluo™ 488 anti-mouse IgG (1:1000, Cat. No. A55058., Invitrogen), DAPI (1:100, Cat. No.C1006, Beyotime) for nuclear staining, and Alexa Fluor™ 555 Phalloidin (1:400, Cat. No. A34055, Invitrogen) for visualizing the cytoskeleton morphology. Immunofluorescence was visualized by confocal microscopy (Leica Stellaris 5, 63× objective). The subcellular localization of POU3F4 WT and variant proteins was assessed by immunofluorescence and quantified using ImageJ software (version 1.54p). The fluorescence images were captured at ×63 magnification using a confocal microscope. For each condition, at least 12 cells were analysed across three independent biological replicates, sampled from multiple fields of view. Quantification was performed in a blinded manner with respect to construct identity. Regions of interest corresponding to the nucleus (DAPI-stained) and cytoplasm were manually defined for each cell. The intensity of Myc-tag fluorescence within each region was measured, and the nuclear overlap coefficient was calculated for each cell. WT cells were used as the reference, with the mean value normalised to 1.0. For statistical analysis, the mean value from each biological replicate was used as the unit of analysis. Comparisons between each variant and its matched rescue construct were performed using two-way ANOVA followed by Šidák’s multiple-comparison test. Data are presented as mean ± SD. Effect sizes are reported as mean differences between variant and rescue conditions.

For luciferase reporter assays, the human *POU3F4* promoter region (−482 to +25 relative to the transcription start site) was cloned into the pGL3-basic vector (Cat. No. P0193, MiaolingBio, China) upstream of the firefly luciferase coding sequence. This promoter fragment was selected based on prior functional characterization demonstrating multiple *POU3F4* binding sites and transcriptional activity within the −457 to +22 region, suggesting autoregulatory function (PMID: 8793109). The same promoter construct has been used in subsequent *POU3F4* transcriptional studies(1, 2), enabling comparability across experiments. HEK293T cells were co-transfected with the promoter–luciferase reporter plasmid, wild-type, variant, or rescued *POU3F4* expression plasmids, and a Renilla luciferase pRL-CMV control vector (Cat. No. P0196, MiaolingBio, China) for normalization of transfection efficiency. 24 hours after transfection, firefly and Renilla luciferase activities were measured using the Dual-Luciferase Reporter Assay System (Cat. No. E1910, Promega). Firefly luciferase activity was normalized to Renilla luciferase activity. Each experiment was performed in triplicate. Statistical analyses for luciferase assays were performed using R software (version 4.4.2). Differences between groups were evaluated using two-tailed Student’s t-test. A p-value <0.05 was considered statistically significant.

To explore the structural impact of stop-loss variants, three-dimensional models of wild-type and extended POU3F4 proteins were generated using AlphaFold2. Structural models were aligned based on the POU functional domain backbone to enable comparison of conformational differences. The spatial proximity between the C-terminal extension sequences and the predicted nuclear localization signal (NLS; residues 275–286) was evaluated by calculating minimal Cα–Cα distances. Visualization and structural measurements were performed using PyMOL (version 4.6.0). Nuclear localization signal sequence predicted by NLS Mapper (<https://nls-mapper.iab.keio.ac.jp/cgi-bin/NLS_Mapper_form.cgi>).

**Reagent Validation**

**1. Cell line validation**

HEK293T (RRID: CVCL_0063) and HeLa (RRID: CVCL_0030) cell lines were obtained from the Cell Bank of the Chinese Academy of Sciences (Shanghai, China).

The identities of the cell lines were authenticated by the provider using short tandem repeat (STR) profiling. According to the supplier’s quality control standards, all cell lines were confirmed to be free of mycoplasma contamination prior to distribution.

Cells were used within a limited number of passages and maintained under recommended culture conditions.

**2. Antibody validation**

All antibodies used in this study are commercially available and were used according to the manufacturers’ instructions. Supplier information and catalogue numbers are provided in the Supplemental Methods section to ensure reproducibility.

The specificity and performance of these antibodies have been validated by the manufacturers and are supported by prior use in published studies. No custom or in-house generated antibodies were used in this study.

**Gene‑specific Adaptations of ACMG‑AMP Classification Criteria for *POU3F4* Gene**

Among the 28 ACMG/AMP evidence criteria, 13 were modified with respect to their utilization and/or strength level for *POU3F4*. Two criteria (PM3 and BS2) were deemed not applicable due to the X-linked inheritance pattern. Four additional criteria (PP2, PP5, BP1, and BP6) had previously been classified as not applicable by the ClinGen Hearing Loss Variant Curation Expert Panel(3). Importantly, PM1 and PP4 were re-specified based on quantitative analyses derived from the CDGC cohort, providing gene-specific calibration of domain enrichment and genotype–phenotype concordance.

**Null variant in a gene where loss-of-function is a known mechanism of disease (PVS1)**

Haploinsufficiency is a well-established disease mechanism for *POU3F4* in DFNX2-related hearing loss(4). Although the constraint metrics for this gene are currently unavailable in gnomAD, our previously published gene-disease curation data demonstrated that *POU3F4* lies within a quadrant in which both the proportion of PTVs classified as P/LP and the proportion of P/LP variants that are PTVs exceed 50%, strongly supporting loss-of-function as the primary pathogenic mechanism(5). Therefore, PVS1 is applicable to putative loss-of-function variants, nonsense, frameshift, and large deletions (canonical splice-site variants are not applicable given the single-exon structure).

Importantly, *POU3F4* consists of a single coding exon and encodes a 361-amino-acid transcription factor containing two well-defined functional domains: the POU-specific domain (aa 181–255) and the POU homeodomain (aa 278–340), both essential for DNA binding and transcriptional regulation(6). Truncating variants that disrupt these domains have been shown to markedly reduce transcriptional activity. Pathogenic truncating variants reported to date are broadly distributed across the coding sequence (aa 22–331), without clustering, consistent with a classical loss-of-function mechanism rather than region-specific effects. Furthermore, no hemizygous loss-of-function variants have been observed in gnomAD. Although the general PVS1 framework recommends downgrading predicted truncating variants in single-exon genes due to the absence of nonsense-mediated decay, we consider that, for *POU3F4*, truncating variants occurring upstream of amino acid 340 (last amino acid of POU homeodomain) are expected to abolish critical functional domains and result in true loss-of-function irrespective of NMD. Therefore, we propose that such variants warrant full-strength PVS1. In contrast, truncating variants occurring between amino acids 341 and 361 lie beyond the final functional domain, and currently lack sufficient evidence of strength; PVS1 should not be automatically applied; strength should be determined based on functional or segregation evidence.

**Assessment of Variant Minor Allele Frequency (BA1, BS1, PM2)**

Allele frequency thresholds for *POU3F4* were derived using a framework analogous to that applied by the ClinGen Variant Curation Expert Panel for the X-linked gene *SLC6A8*(7), which is also associated with an early-onset condition with complete penetrance in hemizygous males. PM2 was applied at the Supporting level (PM2_Supporting) in accordance with ClinGen SVI recommendations.

The allele frequency calculator (<http://cardiodb.org/allelefrequencyapp>) was used to estimate the maximum credible population allele frequency based on disease prevalence, maximum genetic contribution, maximum allelic contribution, and penetrance. In the absence of precise population-based prevalence data for DFNX2, prevalence was conservatively estimated using a stepwise approach: congenital hereditary hearing loss occurs in approximately 1 in 1,000 individuals, X-linked forms account for ~1% of cases, and *POU3F4* represents the majority of X-linked non-syndromic hearing loss. Based on these assumptions, the estimated prevalence of *POU3F4*-related DFNX2 is approximately 1 in 100,000. Penetrance was set at 0.5 to account for heterozygous female carriers who may be asymptomatic or only mildly affected and therefore under-ascertained.

Given the 100% penetrance in hemizygous males, the presence of hemizygous individuals in population databases was incorporated into threshold determination. Based on these assumptions, thresholds were defined as follows: PM2_Supporting at MAF <0.002% with zero hemizygotes observed in gnomAD; BS1 at MAF ≥0.02% (or ≥5 hemizygotes); and BA1 at MAF ≥0.2% (or ≥10 hemizygotes).

**Increased variant prevalence in cases versus controls (PS4)**

Due to the rarity of pathogenic variants in *POU3F4* and the practical infeasibility of conducting adequately powered ancestry-matched case–control studies for individual variants, a proband-counting framework was adopted for PS4 specification. This approach is consistent with the ClinGen Hearing Loss Expert Panel recommendations.

Application of PS4 requires that the variant meets PM2_Supporting and that the reported individuals exhibit a phenotype highly consistent with DFNX2, including characteristic inner ear malformations (e.g., incomplete partition type III) and hearing loss. Only unrelated probands are counted toward PS4 evidence.

The following thresholds are recommended:

PS4_Supporting: ≥2 unrelated male probands with a consistent DFNX2 phenotype

PS4_Moderate: ≥6 unrelated male probands with a consistent DFNX2 phenotype

PS4 (Strong): ≥15 unrelated male probands with a consistent DFNX2 phenotype

Female heterozygous individuals may contribute supporting evidence when phenotype is well documented; however, they are not included in the primary proband count due to variable expressivity and incomplete penetrance in females.

**Mutational hot spot or well-studied functional domain without benign variation (PM1)**

Pathogenic missense variants in *POU3F4* are strongly enriched within the POU-specific domain (aa 181–255) and POU homeodomain (aa 278–340), both of which are critical for DNA binding and transcriptional regulation. All reported pathogenic missense variants localize within these domains, whereas the majority of hemizygous missense variants observed in gnomAD are located outside these regions. We previously demonstrated missense variants located in POU3F4 domains fulfilled the supporting level(5).

Paralog conservation analysis across 15 POU family members further demonstrated significant enrichment of pathogenic missense variants at highly or moderately conserved residues. Variants affecting conserved residues within these domains yielded a positive likelihood ratio (LR+) of 32.97 (95% CI 10.65–101.94), exceeding the threshold for moderate-level PM1 evidence as defined by Tavtigian et al(8).

Accordingly, PM1 is specified for *POU3F4* as follows:

**PM1_Moderate**: Missense variant located within the POU-specific or POU homeodomain AND affecting a highly or moderately conserved residue based on paralog conservation analysis.

**PM1_Supporting**: Missense variant located within the POU-specific or POU homeodomain but not meeting conservation criteria.

PM1 should not be applied to variants located outside these functional domains.

**Patient’s phenotype is highly specific for a disease with a single genetic aetiology (PP4)**

PP4 was specified based on the strong genotype–phenotype correlation between *POU3F4* pathogenic variants and incomplete partition type III (IP-III) malformation. Among 28 individuals with radiologically confirmed IP-III in our cohort, 27 harbored pathogenic or likely pathogenic *POU3F4* variants, yielding a genotype–phenotype concordance of 96.4% (95% CI, 81.6%–99.9%). Despite the limited sample size, the lower bound of the confidence interval remained high, supporting substantial locus specificity.

IP-III is a highly characteristic inner ear malformation that is rarely associated with other genetic etiologies, supporting strong locus homogeneity. Consistent with ClinGen guidance for disorders with high diagnostic yield and gene-specific phenotypic specificity, PP4 is specified at the Strong level for individuals with radiologically confirmed IP-III and a clinical presentation consistent with DFNX2. PP4 should not be applied in cases of nonspecific hearing loss without characteristic radiologic findings.

**Multiple segregations of a variant with phenotype in affected family members (PP1, BS4)**

Segregation evidence (PP1, BS4) for *POU3F4* was specified following ClinGen guidance for X-linked disorders and adapted from the *SLC6A8* framework(7). Segregation of a variant with disease within a family increases the likelihood that the variant is causative, and the strength of evidence correlates with the number of informative meioses observed.

For *POU3F4*, affected hemizygous males carrying the variant should be counted toward segregation evidence. Heterozygous females with hearing loss or imaging findings consistent with DFNX2 may also be counted as affected individuals.

Unaffected variant-negative males within the family may be counted as segregation events. However, heterozygous females without clinical manifestations should not be counted as non-segregation events, given the variable expressivity and reduced penetrance in females. Similarly, absence of disease in female carriers should not be used as evidence against pathogenicity (i.e., should not be used for BS4).

Strength assignment for PP1 should follow the ClinGen quantitative segregation framework, with increasing strength corresponding to the number of informative meioses observed.

BS4 should only be applied when a variant fails to segregate in clearly affected hemizygous males within a well-characterized pedigree.

**De novo events (PS2, PM6)**

De novo evidence (PS2, PM6) for *POU3F4* was specified following ClinGen guidance for X-linked disorders and adapted from the *SLC6A8* framework. For *POU3F4*, PS2 (de novo) was applied if there was an affected male with a variant and a non-carrier mother and a confirmed maternity. For an affected female both paternity and maternity confirmation was required. For an apparently de novo variant in a male, without confirmation of maternity, or in a female without confirmation of maternity and paternity, PM6 was applied. Consistent with ClinGen de novo recommendations, the strength of PS2/PM6 may be adjusted based on phenotypic specificity. Full strength should be applied only when the phenotype is highly specific for DFNX2, particularly in the presence of radiologically confirmed incomplete partition type III (IP-III). In cases with less specific hearing loss phenotypes, de novo evidence may warrant strength downgrading.

**Functional evidence supportive of a damaging effect or no effect (PS3, BS3)**

Evaluation of functional data for *POU3F4* variants should follow the ClinGen Sequence Variant Interpretation (SVI) framework for application of PS3 and BS3(9). Consistent with this recommendation, the VCEP specifies the types of experimental assays that are appropriate for assessing *POU3F4* variants based on its established loss-of-function disease mechanism, as shown in Figure1.

Assays deemed appropriate include complementary DNA (cDNA) analyses assessing transcript consequences relevant to loss of function, and in vitro engineered systems evaluating transcriptional activity or DNA binding, such as luciferase reporter assays and electrophoretic mobility shift assays (EMSA) or chromatin immunoprecipitation (ChIP) assays. In vivo knock-in models that faithfully recapitulate the DFNX2 phenotype may also be considered when appropriately validated.


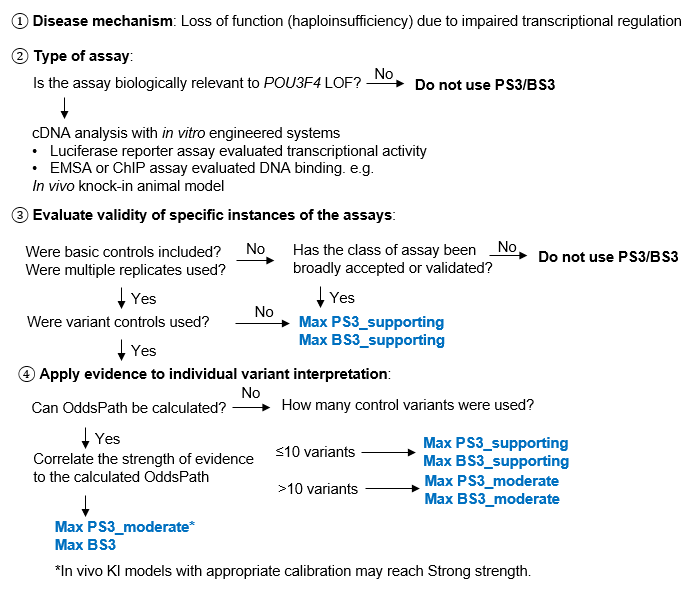
Functional studies deemed inappropriate for application of PS3 or BS3 include assays measuring nonspecific downstream gene expression changes or indirect cellular or morphological phenotypes that do not directly assess the transcriptional regulatory function of POU3F4.

**Figure 1: Flowchart for the adapted PS3/BS3 criteria for functional evidence in *POU3F4*.**
The decision framework follows the ClinGen Sequence Variant Interpretation (SVI) recommendations for application of PS3 and BS3, with gene-specific adaptations reflecting the established loss-of-function disease mechanism of *POU3F4*.

**Reference**

1. Lee HK, Song MH, Kang M, Lee JT, Kong KA, Choi SJ, et al. Clinical and molecular characterizations of novel POU3F4 mutations reveal that DFN3 is due to null function of POU3F4 protein. Physiological genomics. 2009;39(3):195-201.

2. Bernardinelli E, Roesch S, Simoni E, Marino A, Rasp G, Astolfi L, et al. Novel POU3F4 variants identified in patients with inner ear malformations exhibit aberrant cellular distribution and lack of SLC6A20 transcriptional upregulation. Frontiers in molecular neuroscience. 2022;15:999833.

3. Oza AM, DiStefano MT, Hemphill SE, Cushman BJ, Grant AR, Siegert RK, et al. Expert specification of the ACMG/AMP variant interpretation guidelines for genetic hearing loss. Hum Mutat. 2018;39(11):1593-613.

4. Dang J, Bian P, Chen C, Chen C, Shan W, Cai L, et al. Impact of POU3F4 mutation on cochlear development and auditory function. Cell communication and signaling : CCS. 2025;23(1):121.

5. Cheng H, Wang X, Zhong M, Geng J, Li W, Pei K, et al. GDC: Integration of Multi-Omic and Phenotypic Resources to Unravel the Genetic Pathogenesis of Hearing Loss. Advanced Science. 2025;12(29):2408891.

6. Choi BY, Kim DH, Chung T, Chang M, Kim EH, Kim AR, et al. Destabilization and mislocalization of POU3F4 by C-terminal frameshift truncation and extension mutation. Hum Mutat. 2013;34(2):309-16.

7. Goldstein J, Thomas-Wilson A, Groopman E, Aggarwal V, Bianconi S, Fernandez R, et al. ClinGen variant curation expert panel recommendations for classification of variants in GAMT, GATM and SLC6A8 for cerebral creatine deficiency syndromes. Molecular genetics and metabolism. 2024;142(1):108362.

8. Tavtigian SV, Greenblatt MS, Harrison SM, Nussbaum RL, Prabhu SA, Boucher KM, et al. Modeling the ACMG/AMP variant classification guidelines as a Bayesian classification framework. Genetics in medicine : official journal of the American College of Medical Genetics. 2018;20(9):1054-60.

9. Brnich SE, Abou Tayoun AN, Couch FJ, Cutting GR, Greenblatt MS, Heinen CD, et al. Recommendations for application of the functional evidence PS3/BS3 criterion using the ACMG/AMP sequence variant interpretation framework. Genome medicine. 2019;12(1):3.
