# Supplementary material for: Optimising POU3F4 variant interpretation through gene-specific evidence in X-linked hearing loss
Source: eBioMedicine. 2026 May 29;128:106318. doi: 10.1016/j.ebiom.2026.106318 (PMC13242011; doi:10.1016/j.ebiom.2026.106318)
Supplement: Reagent validation [file mmc4.pdf]

Cell line Authentication Report

Date: 2026-3-30

1. Sample information

Cell line name: HeLa

2. methods:

1. Genomic DNA was extracted using Magpure tissue&blood DNA LQ kit.
2. Samples, together with positive and negative control were amplified using thermo cycler.
3.Amplified products were processed using ABI3730xl Genetic Analyzer.
4.Data were analyzed using GeneMapper6.0 software and then compared with the Cellosaurus database for reference matching.

3. Results

Electropherogram

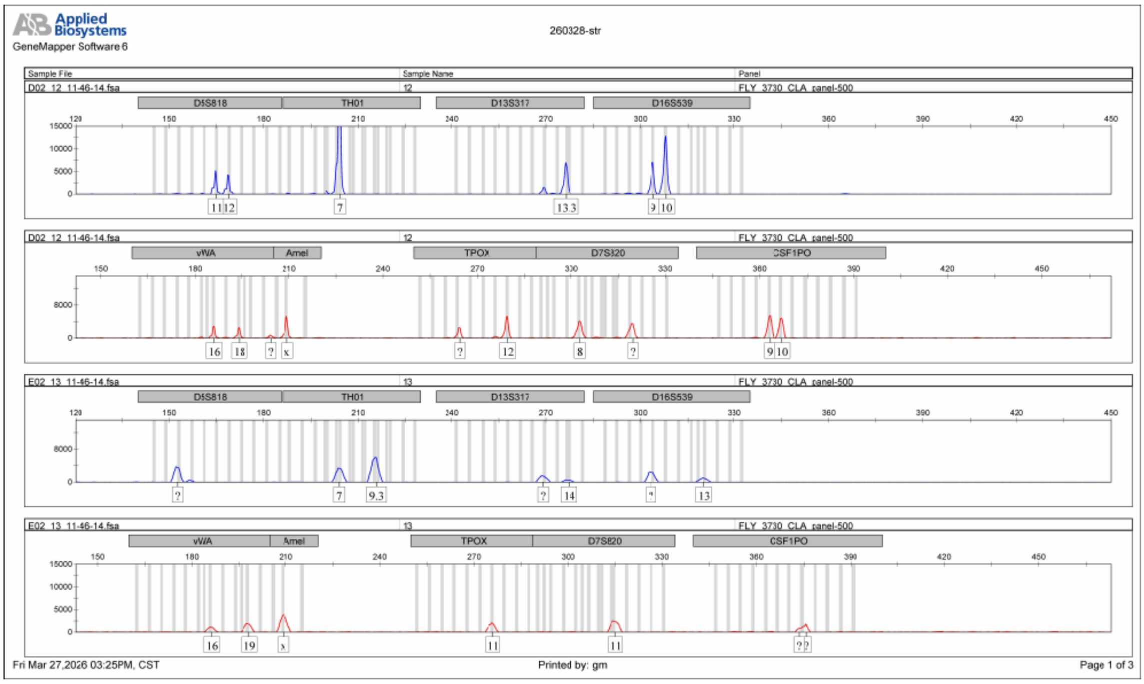

STR Profile

Marker	Sample			Cellosaurus		
	Allele1	Allele2	Allele3	Allele1	Allele2	Allele3
D5S818	11	12		11	12	
TH01	7			7		
D13S317	12	13.3		12	13.3	
D16S539	9	10		9	10	
vWA	16	18		16	18	
TPOX	8	12		8	12	
D7S820	8	12		8	12	
CSF1PO	9	10		9	10	

Amel	X	X

Comparison results

Accession	Name	Nº Markers	Score	Amel	CSF1PO	D2S1338	D3S1358	D5S818	D7S820	D8S1179	D13S317	D16S539	D18S51	D19S433	D21S11	FGA	Penta D	Penta E	TH01	TPOX	vWA
NA	Query	NA	NA	X	9,10			11,12	8,12		12,13.3	9,10							7	8,12	16,18
CVCL_0030 *Best*	HeLa	8	100.00%	X	9,10	17	15,18	11,12	8,12	12,13	12,13.3	9,10	16	13,14	27	18,21	8	7,17	7	8,12	16,18
CVCL_0030 *Worst*	HeLa	8	93.33%	X	9,10	17	15,18	11,12	8,12	12,13	13,13.3	9,10	16	13,14	27,28	18,21	8,15	7,17	7	8,12	16,18
CVCL_1276 *Best*	HeLa 229	8	100.00%	X	9,10	17	15,18	11,12	8,12	12,13	12,13.3	9,10	16	13,14	27,28	18,21	8,15	7,17	7	8,12	16,18
CVCL_1276 *Worst*	HeLa 229	8	89.66%	X	9,10	17	15,18	11,12	8,12	12,13	14	9,10	16	13,14	27,28	18,21	8,15	7,17	7	8,12	16,18
CVCL_ZM02	HeLa H2B-GFP [USA 1997]	8	100.00%	X	9,10		15,18	11,12	8,12	12,13	12,13.3	9,10	16		27,28	21	8,15	7,17	7	8,12	16,18

4 Conclusion

1. The STR results showed that there were no four alleles in each locus, and no cross contamination of human cells was found in the cell lines.
2. The percent match between the sample and the database (https://web.expasy.org/cellosaurus/) profile is 100%, the cell name is HeLa**.**

Report issuing unit：Genomics Center, Core Facilities of West China Hospital

Operator: **liyuan Fang**

Cell line Authentication Report

Date: 2026-3-30

1. Sample information

Cell line name: HEK293

2. methods:

1. Genomic DNA was extracted using Magpure tissue&blood DNA LQ kit.
2. Samples, together with positive and negative control were amplified using thermo cycler.
3. Amplified products were processed using ABI3730xl Genetic Analyzer.
4. Data were analyzed using GeneMapper6.0 software and then compared with the Cellosaurus database for reference matching.

3. Results

Electropherogram

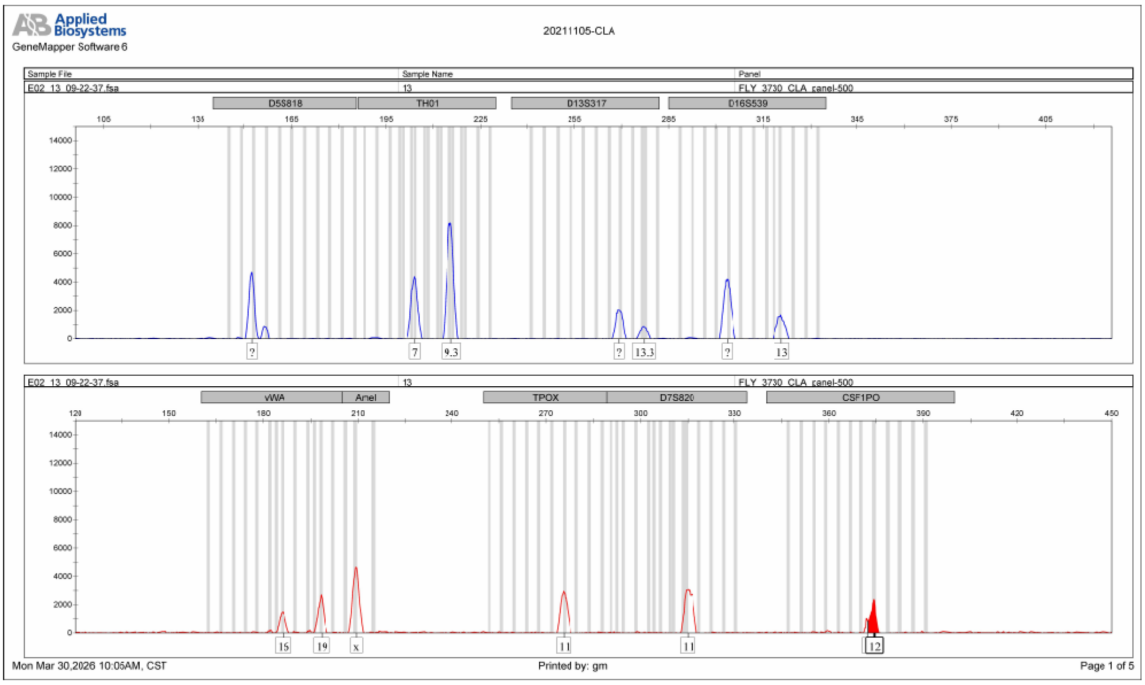

STR Profile

Marker	Sample			Cellosaurus		
	Allele1	Allele2	Allele3	Allele1	Allele2	Allele3
D5S818	8	9		8	9	
TH01	7	9.3		7	9.3	
D13S317	12	14		12	14	
D16S539	9	13		9	13	
vWA	16	19		16	19	
TPOX	11			11		
D7S820	11			11	12	
CSF1PO	12			11	12	

Amel	X	X

Comparison results

Accession	Name	N° Markers	Score	Amel	CSF1PO	D2S1338	D3S1358	D5S818	D7S820	D8S1179	D13S317	D16S539	D18S51	D19S433	D21S11	FGA	Penta D	Penta E	TH01	TPOX	vWA
NA	Query	NA	NA	X	12			8,9	11		12,14	9,13							7,9.3	11	16,19
CVCL_0045 *Best*	HEK293	8	96.30%	X	11,12	19	15,17	8,9	11	12,14	12,14	9,13	17	15,18	28,30.2	23	9,10	7,15	7,9.3	11	16,19
CVCL_0045 *Worst*	HEK293	8	84.62%	X	11,12	19	15,17	8,9	11,12	12,14	12	9	18	18	28,30.2	23	9,10	7,15	7,9.3	11	16,19
CVCL_0063 *Best*	HEK293T	8	96.30%	X	11,12	19	15,16,17	8,9	11	12,14	12,14	9,13	17,18	18	28,30.2	23	9,10	7,15	7,9.3	11	16,19
CVCL_0063 *Worst*	HEK293T	8	92.31%	X	11,12	19	15,17	8,9	11	12,14	12	9,13	17,18	18	28,30.2	23	9,10	7,15	7,9.3	11	16,19

4 Conclusion

3. The STR results showed that there were no four alleles in each locus, and no cross contamination of human cells was found in the cell lines.
4. The percent match between the sample and the database (https://web.expasy.org/cellosaurus/) profile is 96%, the cell name is HEK293**.**

Report issuing unit：Genomics Center, Core Facilities of West China Hospital

Operator: **liyuan Fang**

Report of Mycoplasma Dection

Date: April 1st, 2026

1. **Sample**

   Sample Name: HEK293T, label as '293T'; Hela, label as 'Hela'

2. **Method and procedure**

3. **Results**

   293T: Contamination of mycoplasma was not found in the sample.

   Hela: Contamination of mycoplasma was not found in the sample.

   Water was used as negative control.

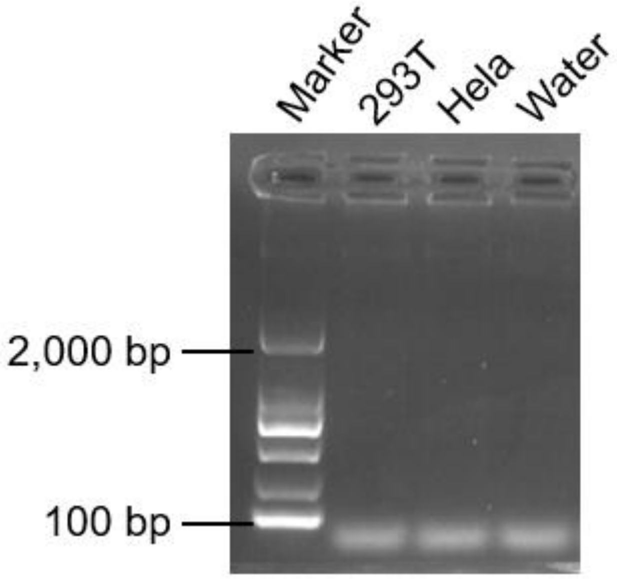

Operator: Yu Huang
