# Supplementary figures and images for: Optimising POU3F4 variant interpretation through gene-specific evidence in X-linked hearing loss
Source: eBioMedicine. 2026 May 29;128:106318. doi: 10.1016/j.ebiom.2026.106318 (PMC13242011; doi:10.1016/j.ebiom.2026.106318)

Full unedited gel for Figure 4 C

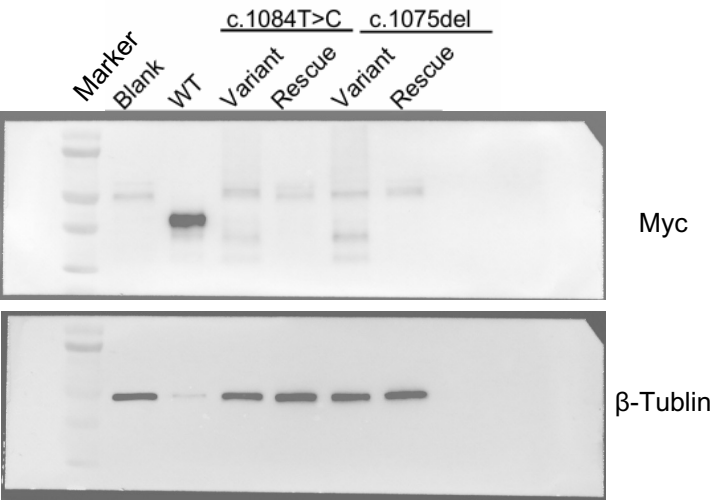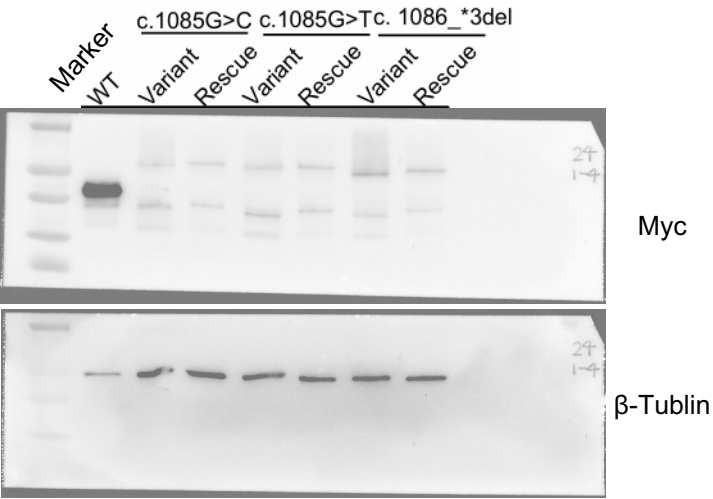

Full unedited gel for Figure S10 A

Full unedited gel for Figure S10 B

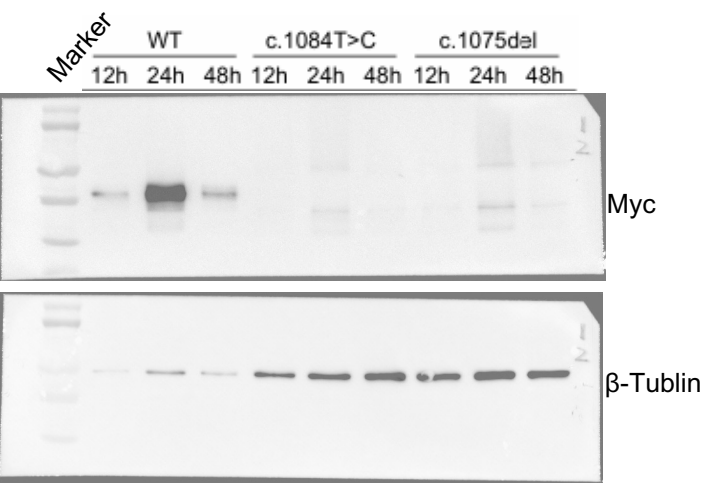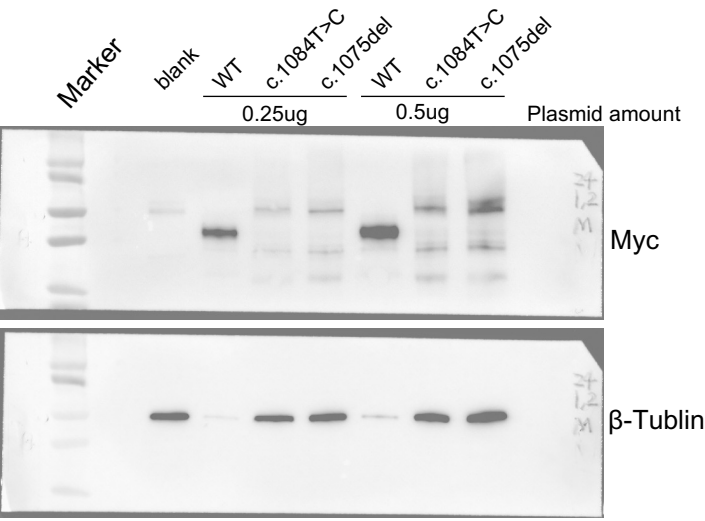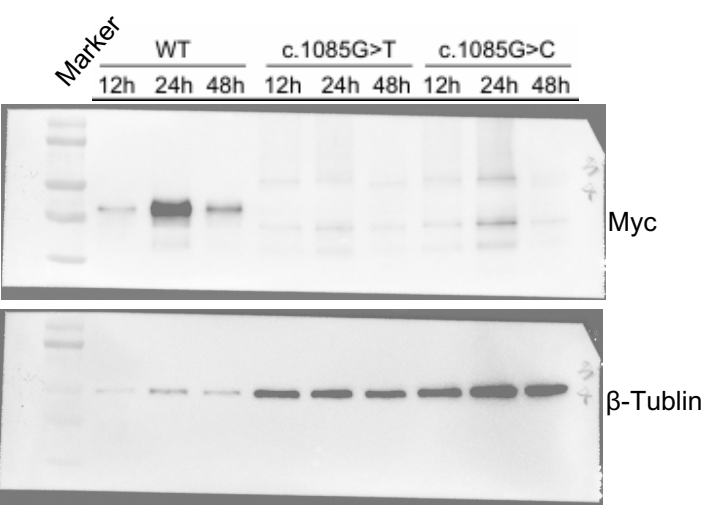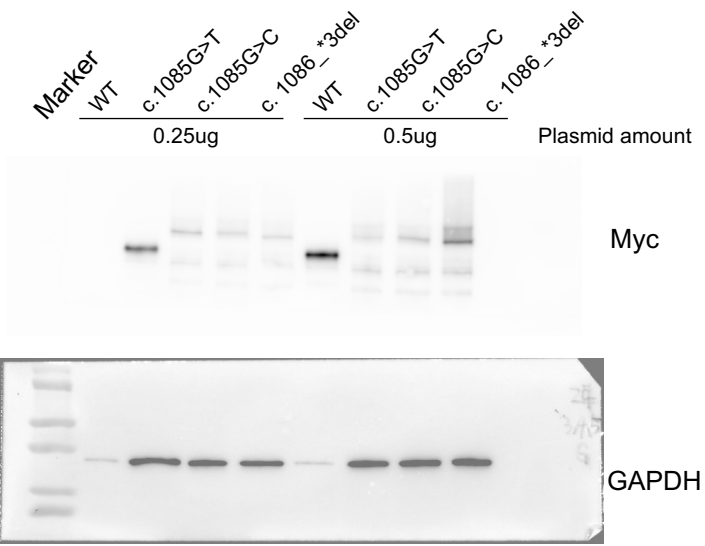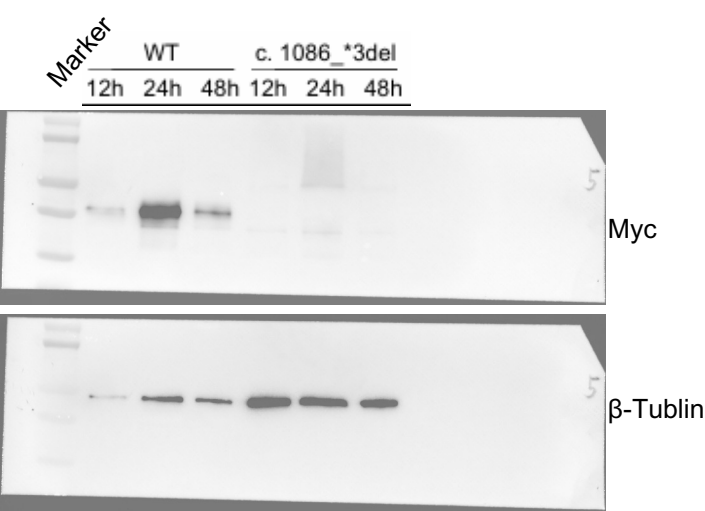

Supplement: Full unedited gel [file mmc5.pdf]
